# Bayesian Identification and Estimation of Growth Mixture Models

**DOI:** 10.1017/psy.2025.11

**Published:** 2025-04-07

**Authors:** Xingyao Xiao, Sophia Rabe-Hesketh, Anders Skrondal

**Affiliations:** 1 BSE, University of California, Berkeley, CA, USA; 2 CEFH, Norwegian Institute of Public Health, Oslo, Norway; 3 CREATE & CEMO, University of Oslo, Oslo, Norway

**Keywords:** degenerate nonidentifiability, distinguishability index, finite mixture model, Hamiltonian Monte Carlo, latent class model

## Abstract

This article addresses problematic behaviors of Markov chain Monte Carlo (MCMC) methods for finite mixture models due to what we call degenerate nonidentifiability. We discuss the reasons for these behaviors, propose diagnostics to detect them, and show through simulations that using more informative priors than the vague defaults can mitigate the problems in growth mixture models (GMMs). Our motivating example is an application of GMMs to data from the National Longitudinal Survey of Youth (NLSY) to examine heterogeneity in the development of reading skills in children aged 6–14. We also suggest ways of describing and visualizing within-class heterogeneity in GMMs, provide a literature review of likelihood identification and Bayesian identification, propose a viable definition of Bayesian identification for latent variable models based on the marginal likelihood (integrated over the latent variables), and give a brief didactic description of Hamiltonian Monte Carlo (HMC) as implemented in Stan.

## 1 Introduction

The ability to read is fundamental for learning and for effective participation in society and the workplace. It is therefore not surprising that research has been concerned with the development of children’s reading skills over time. In this article, we will use data from the National Longitudinal Survey of Youth (NLSY) to address the question of whether children fall into subgroups characterized by different types of learning trajectories for reading recognition.

The standard approach for addressing such research questions is the use of growth mixture models (GMMs) which are finite mixtures of growth curve models (e.g., Muthén, 2002; Muthén & Muthén, 2000; Muthén & Shedden, 1999). In GMMs, each subpopulation or class has its own mean growth trajectory, often linear or quadratic in time, with intercepts and slopes that vary between individuals. The covariance matrix for intercepts and slopes is usually also class-specific although an equality constraint is sometimes imposed to address convergence issues (e.g., McNeish & Harring, 2020). Individuals’ class membership and the values of their intercepts and slopes can be viewed as latent variables, and we will use the term “marginal likelihood” to refer to the likelihood obtained by marginalizing over these latent variables.

Bayesian estimation is increasingly used for GMMs, with researchers employing tools such as Mplus (Muthén & Muthén, 1998–2017) or general Bayesian languages such as BUGS or OpenBUGS (Lunn et al., 2012), JAGS (Plummer, 2017), and Stan (Stan Development Team, 2024). In this article, we use the popular Stan software which implements Hamiltonian Monte Carlo (HMC) methods (Betancourt & Girolami, 2015; Hoffman & Gelman, 2014).

Similar to other finite mixture or latent class models, estimation and inference are challenging for GMMs. For Bayesian estimation, the existing literature has addressed issues such as label switching (e.g., Diebolt & Robert, 1994) and multimodality (e.g., Yao et al., 2022), but to our knowledge there is no general treatment of identifiability issues and their implications for estimation.

We will argue that Bayesian identifiability is essentially the same as likelihood identifiability and provide an overview of different kinds of likelihood identifiability, such as global, local, weak, and empirical identifiability. Importantly, we will show that the *marginal* likelihood is appropriate for investigating identifiability in latent variable models.

Special identifiability issues arise for finite mixture models such as GMMs. Labeling nonidentifiability is due to the invariance of the marginal likelihood to permutations of the component/class labels and has been thoroughly addressed in the literature because it leads to label switching. The other issues are related to degeneracies whereby a *K*-class model becomes equivalent to a model with fewer than *K* classes. Such degeneracies are due to parameter vectors satisfying certain constraints. Several parameter vectors, each satisfying a distinct set of constraints that lead to degeneracy, can be observationally equivalent in the sense that they produce the same marginal likelihood, leading to degenerate nonidentifiability. Additionally, the model is locally nonidentified at degenerate parameter values. This can happen in two ways. First, when one or more class probabilities (or mixing proportions) are zero, the class-specific parameters for the corresponding class or classes are not identified. Second, when the parameters of two (or more) classes are identical, the corresponding class probabilities are not separately identified—only their sum. The first issue turns out to be problematic for MCMC estimation because it is self-perpetuating in the sense that sampling a tiny class probability will lead to sampling of arbitrary class-specific parameters for that class, which in turn will lead to sampling of a tiny class probability when these class-specific parameters are incompatible with the data. We refer to such sequences of persistent sampling of tiny class probabilities as “stuck” sequences when the sampled values remain unchanged and as “miniscule-class” sequences when the class-probability parameter(s) fluctuate just above zero with a small variance.

The main contributions of the article are as follows: (1) using GMMs to analyze and classify development of children’s reading recognition based on NLSY data and proposing methods to visualize and describe within-class heterogeneity in such models, (2) providing a review of the literature on Bayesian identification and proposing a viable definition for latent variable models, (3) giving a brief didactic description of HMC estimation methods, (4) demonstrating problematic behavior of MCMC methods due to identifiability issues, (5) suggesting diagnostics and demonstrating their usefulness for detecting problematic behavior, and (6) recommending the use of weakly informative priors to mitigate the problems and investigating their performance through a simulation study. Data and code for generating the graphs and diagnostics presented here are available on https://github.com/DoriaXiao/BayesianIdentification.

In the next section, we analyze data from NLSY to motivate and introduce GMMs and interpret estimates as preparation for subsequent sections on identifiability, estimation, and convergence issues.

## 2 Bayesian growth mixture modeling of reading recognition

### 2.1 Goals of analysis

Our goal is to identify subpopulations of children characterized by distinct mean growth trajectories of reading recognition and distinct types of heterogeneity in their growth trajectories from age 6 to 14. Applications of growth mixture modeling to reading include Kaplan (2002), Bilir et al. (2008), Boscardin et al. (2008), Pianta et al. (2008), Grimm et al. (2010), and Grimm et al. (2017).

Some of these studies focus on narrower age ranges than ours and are therefore less comparable to our study. For example, Bilir et al. (2008) considered older students in 6th–9th grade (ages 12–15), whereas Kaplan (2002) and Boscardin et al. (2008) considered early reading development over a 1.5-year and 2-year period, respectively, starting at the end of kindergarten/beginning of first grade (about six years old).

More similar to our study, Pianta et al. (2008) analyzed reading scores from the National Institute of Child Health and Human Development (NICHD) Study of Early Child Care and Youth Development (SECCYD) for children aged 54 months (4.5 years) in Wave 1 and followed up in first, third, and fifth grade (11 years old). Pianta et al. (2008) fitted a two-class GMM and identified “fast readers” (30% of children) whose average trajectory grows fast initially and then decelerates and “typical readers” (70% of children) who start lower, on average, and grow approximately linearly, remaining below fast readers through 5th grade. Similar average latent trajectory curves were found by Grimm et al. (2010) who analyzed data from the Early Childhood Longitudinal Study—Kindergarten (ECLS-K) on children from the end of Kindergarten to the end of eighth grade, but only 6% of children fell in their “early reader” class, with the remainder in the “normative” class. That paper used nonlinear (Gompertz) growth curves, whereas a subset of the same data is analyzed using classic GMMs by Grimm et al. (2017). Their three-class solution identified a class (54% of students) whose mean trajectory starts at the lowest point among the classes at the end of Kindergarten and shows steady growth through fifth grade followed by a slight deceleration. The trajectory of the smallest class (11% of students) starts higher and is nearly parallel to that of the largest class, whereas the mid-sized class (35% of students) starts only slightly higher than the largest class and grows considerably faster initially, catching up to the smallest class by about 3rd grade.

### 2.2 Subjects and measures

We use data from the National Longitudinal Survey of Youth (NLSY) provided by Curran (1997) and previously analyzed by Curran et al. (2004), Bollen & Curran (2006), McNeish & Harring (2020), and others. The sample includes one child per mother, aged between six and eight years in the first wave in 1986 and with complete data in 1986 for the variables considered by Curran (1997). The children were assessed every two years for four waves of data until 1992. Our analysis focuses on the scores from the reading recognition subtest of the Peabody Individual Achievement Test (PIAT). This subtest assesses word recognition and pronunciation ability which are considered essential components of reading ability. There were 405 children in Wave 1. Among them, 221 children had reading recognition scores at all four waves, and the average number of reading scores per child was 3.21.

As described by Curran (1997), the reading recognition score in the data is the total raw score on the reading recognition subtest of the PIAT divided by 10. According to the NLSY documentation, the interviewer showed children words to read aloud and rated the pronunciation as correct or incorrect. The same 84 items, sorted in order of increasing difficulty (e.g., item 19 is “run,” item 50 is “guerilla,” and item 84 is “apophthegm,” and the first 18 items are simply numbers and letters, like “1” and “N”), were used for all ages. Which item was first shown to the child (starting item) depended on the child’s PIAT math score. The basal item was established as the lowest item of the highest five consecutive correct responses (proceeding backward from the starting item if necessary). The ceiling item, which was also the last item administered, was the highest item of the lowest seven consecutive responses containing five incorrect responses or errors (in some years the lowest five with consecutive errors). Assuming that children would respond correctly to items lower than (and therefore easier than) the basal item and incorrectly to items higher than the ceiling item, the total raw score was calculated as the ceiling item minus the number of errors between basal and ceiling items.

### 2.3 Bayesian GMM

#### 2.3.1 A GMM for the reading data

The model can be specified by introducing a categorical latent variable 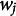 that represents which of *K* latent classes subject *j* belongs to and is sampled from a multinomial distribution with probability parameters 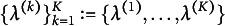 , 

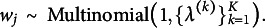

Then the conditional density of the response 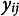 for child *j* at occasion *i*, given class membership 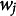 , a random intercept 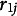 , and a random slope 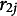 is 

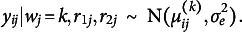

The conditional mean is modeled as a function of the age 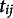 of the child (centered at six years old), 
(1)

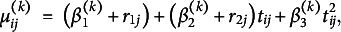

where 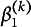 , 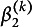 , and 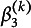 are the mean intercept and slopes of age and age-squared for class *k*. The random intercept and random slope of age are assumed to have a bivariate normal distribution 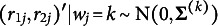 with class-specific covariance matrix 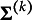 . We include a quadratic term in the conditional mean because we expect the rate of growth in reading recognition to decline as children become more proficient. However, allowing the coefficient of the quadratic term to vary between children would introduce another three variance-covariance parameters for each class and seems overly ambitious because there are at most four observations per child.

The specification so far is sufficient for maximum likelihood estimation. The Bayesian model further has a Dirichlet prior for the class probabilities, with concentration parameters 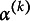 . We set these parameters constant across classes, 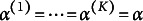 , 

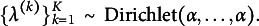

The concentration parameter can be roughly interpreted as adding 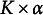 subjects to the data and treating their class membership as known, with 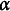 individuals belonging to each class. The greater 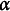 , the stronger our prior belief that the class probabilities are equal. (Gelman et al. (2014, p. 536) refer to 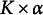 as the “prior sample size.”)

The class-specific random-intercept and random-slope standard deviations, 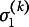 and 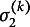 , each have a half-normal or half-Cauchy prior (see, e.g., Gelman, 2006); these are distributions with mean parameter equal to zero and standard deviation or scale parameter 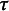 set to a value greater than zero that have been truncated on the left at zero. A small 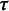 represents a strong belief that the standard deviation is close to zero, whereas a large 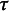 corresponds to a vague prior. The correlation matrix of the random intercepts and slopes is assigned an LKJ (Lewandowski et al., 2009) prior which is proportional to the determinant of the correlation matrix raised to the power 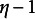 , where 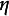 is the shape parameter. In two dimensions with correlation 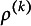 , this corresponds to 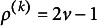 , where *v* has a Beta( 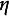 , 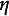 ) distribution on the ( 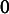 , 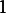 ) interval. With a shape parameter 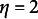 , this prior slightly favors correlations close to zero.

The class-specific mean intercept and slopes of time and time-squared for class *k* have normal priors with zero means and the following variances (written as squared standard deviations): 
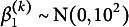
 , 
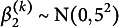
 , and 
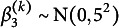
 . Finally, the residual standard deviation, constant across classes, has prior 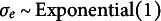 .

In this article, we will use different values of 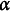 and different priors for 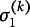 and 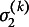 (half-Cauchy versus half-normal and different parameters 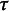 ) and keep all other priors as described here.

We are aware of only one other paper that applies GMMs to these data, namely McNeish & Harring (2020) who use a frequentist approach. Instead of modeling reading as a function of age, they use the survey wave as their time variable even though children’s ages varied between six and eight years old in Wave 1. They also constrained the covariance matrices to be constant across classes, 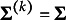 , to achieve convergence for their three-class model. Finally, they only present estimates of the mean growth trajectories and do not provide any information on within-class heterogeneity.

#### 2.3.2 Visualizing and describing within-class heterogeneity

The model allows development trajectories to vary between children within the same class, and the nature of this variation is of scientific interest. However, in papers applying GMMs, typically only the estimated class-specific means are discussed, and variance-covariance parameters for the random effects are often not even reported (see e.g., McNeish & Harring (2020) for a discussion).

Here we introduce some ways to visualize and describe the model-implied within-class heterogeneity. For notational convenience, we drop 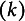 superscripts here, but all results refer to the parameters of a specific class. We also write expressions as if the parameters were known, acknowledging that estimates will be substituted in these expressions.

Let 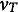 denote the standard deviation of the trajectories for a given class at time 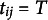 , given by 

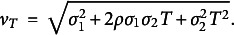

We suggest plotting the mean trajectory together with “50% mid-range” intervals of 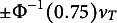 that are expected to contain 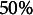 of the middle trajectories at each time-point (where 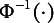 is the inverse standard normal cumulative distribution function).

To convey how the slopes vary, we suggest making statements about the slopes for given values of the intercept. We therefore write the slope for subject *j* as a linear regression on the intercept for the same subject as 

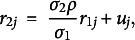

where 

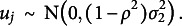



Assuming that 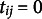 is a starting point of interest, we might then consider individuals who initially deviate from the class-specific mean trajectory by 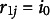 and report how much such individuals would be expected to deviate from the mean trajectory at a later time of interest, 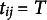 . The expectation of the slope for these individuals is 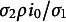 and the expected deviation at time *T* is 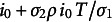 . To avoid specifying an initial deviation, we suggest reporting the percentage change in deviation (conditional on initial deviation) as 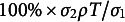 .

We can also consider these deviations from the mean trajectory at times 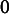 and *T* in standard deviation units (and possibly report the corresponding percentiles within the distributions), with initial standardized deviation given by 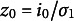 and the expected standardized deviation at time *T* given by 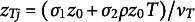 . Finally, we can express how likely it is that someone with initial standardized deviation 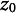 has a positive random slope, 

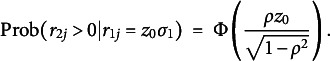



### 2.4 Results

Initially, we used D2C5 priors (Dirichlet with 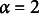 for class probabilities and half-Cauchy with 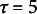 for the standard deviation parameters) but encountered problematic behavior, such as a probability parameter being stuck at zero in some chains. Finding that these issues were related to identifiability inspired the work presented later in the article. In this section, we focus on the model and its application, and choose weakly informative priors to avoid convergence problems.

Specifically, we chose a D10N50 prior combination (Dirichlet with 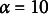 for class probabilities and half-normal with standard deviation 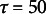 for random intercept and random slope standard deviation parameters), and ran five chains in CmdStan (version 2.30, Stan Development Team, 2021), the shell interface to Stan. Each chain comprised 1,000 iterations after a warmup of 1,000 iterations.

Table 1 shows the Watanabe-Akaike information criterion (WAIC; Watanabe, 2010) and the Pareto-smoothed importance sampling leave-one-out (LOO) estimate, 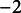 ELPD_LOO, of the same target quantity, computed using the loo package (Vehtari et al., 2016, 2017). As advocated by Merkle et al. (2019), we base these information criteria on the *mixed* predictive distribution (Gelman et al., 1996) which is marginal over the latent variables, here the class membership indicators and the random intercepts and slopes. With this choice, the criteria assess how predictive the models would be for future children like this. See also Xiao (2025) for a discussion and evaluation of these and other information criteria for GMMs. Here both criteria prefer the three-class solution, and we therefore select this solution.

**Table 1:** Information criteria for GMMs with 1–4 classes with D10N50 priors (smallest value for each criterion in italics)

	Number of classes			
Information criterion	1	2	3	4
WAIC	2,988.74	2,919.56	*2,897.10*	2,924.42
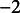 ELPD_LOO	2,988.74	2,919.74	*2,898.96*	2,908.58

**Figure 1:**
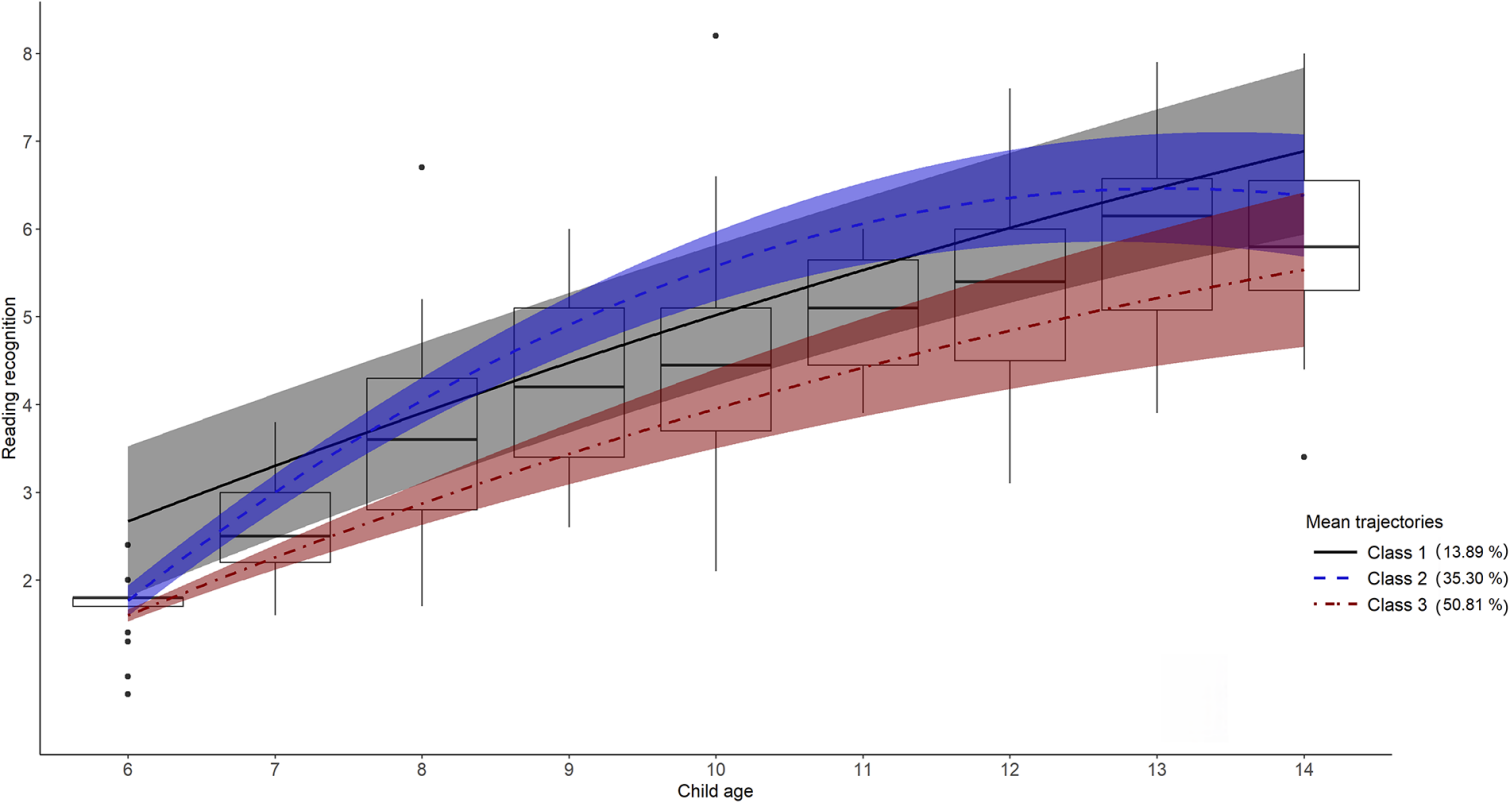
(Color online) Class-specific mean trajectories with shaded 50% mid-range and box-plots of reading scores. *Note*: Class 1 is “Early Bloomers,” Class 2 is “Rapid Catch-Up Learners,” and Class 3 is “Steady Progressors.”

Figure 1 shows class-specific mean trajectories, together with shaded areas representing the 50% mid-range intervals ( 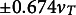 ), as explained in Section 2.3.2. Boxplots of the observed reading scores are also shown. Table 2 presents parameter estimates for this model.

**Table 2:** Estimates for preferred three-class GMM with D10N50 priors

Class	Parameter	Posterior	Posterior	Cred. interval		
		mean	SD	2.50%	97.50%	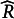
1	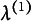	0.139	0.056	0.043	0.260	1.010
	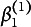	2.668	0.628	1.927	4.215	1.011
	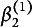	0.648	0.260	0.003	1.024	1.011
	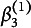	-0.015	0.028	-0.059	0.053	1.008
	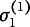	1.269	0.447	0.785	2.293	1.006
	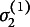	0.156	0.110	0.050	0.298	1.009
	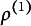	-0.742	0.226	-0.970	-0.141	1.004
2	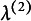	0.353	0.063	0.235	0.479	1.003
	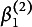	1.765	0.085	1.595	1.930	1.003
	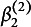	1.329	0.071	1.196	1.475	1.003
	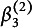	-0.094	0.009	-0.113	-0.077	1.003
	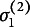	0.258	0.085	0.078	0.421	1.003
	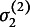	0.119	0.020	0.083	0.162	1.000
	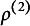	0.399	0.281	-0.178	0.884	1.000
3	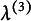	0.508	0.057	0.397	0.619	1.003
	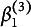	1.597	0.054	1.494	1.703	1.004
	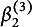	0.685	0.049	0.585	0.778	1.002
	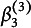	-0.024	0.005	-0.034	-0.013	1.002
	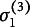	0.102	0.057	0.008	0.218	1.001
	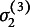	0.159	0.015	0.129	0.189	1.001
	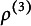	0.485	0.338	-0.384	0.928	1.001
	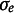	0.462	0.014	0.436	0.490	1.001

We interpret the trajectory classes as follows:

1. **Early Bloomers (14%):** This class starts with a high mean score at age six, exhibiting positive and roughly linear mean growth over time. The latent growth curves vary more at age six than for the other two classes, and there is substantial variation in the slopes (not apparent from the figure) with a negative correlation between intercepts and slopes (estimated as 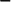 0.742). To elaborate on the within-class heterogeneity as recommended in Section 2.3.2, students whose latent growth curve is two standard deviations below the mean at age six have an 88% chance of growing faster than average, and by age 14, these students are on average only 0.226 standard deviations below the mean. Similarly, two standard deviations above the mean at age six corresponds to an 88% chance of growing more slowly than average and an average of only 0.226 standard deviations above the mean by age 14. Based on their intercepts, students are predicted to deviate 73% less from the mean at age 14 than at age six.
2. **Rapid Catch-Up Learners (35%):** Starting with a lower mean trajectory than the Early Bloomers at age six, on average this class grows rapidly initially, outperforming Early Bloomers for a few years, and then decelerates to become similar to Early Bloomers by about age 13 (the slight decline is a likely artifact of using a quadratic function to approximate the mean growth curve which is unlikely to decline). There is a small amount of heterogeneity in the latent growth trajectories at age six, and these trajectories tend to diverge due to the positive correlation between intercepts and slopes (estimated as 0.399) which implies that there is an 81% chance of growing faster (slower) than average for those whose trajectories lie two standard deviation above (below) the average at age six. Based on their intercepts, students are predicted to deviate 147% more from the class mean at age 14 than at age six, i.e., the deviation is predicted to be about 2.5 times as great.
3. **Steady Progressors (51%):** Characterized by the lowest mean scores at age six, this group showcases an approximately linear growth pattern. While the average gap between Classes 1 and 3 is approximately constant, the relatively large random slope standard deviation (estimated as 0.159) and slope-intercept correlation (estimated as 0.485) results in a fanning out of growth trajectories, leading to considerable overlap with the other two classes by age 14. Based on their intercepts, students are predicted to deviate 605% more from the class mean at age 14 than at age six, i.e., the deviation is predicted to be about seven times as great.

In terms of the mean growth trajectories and class membership probabilities, our estimates are perhaps most similar to the three-class solution of Grimm et al. (2017) who did not name the classes. Our steady progressors (Class 3) seem to be similar in nature to the “typical readers” found by Pianta et al. (2008), whereas our rapid catch-up learners (Class 2) resemble their “fast readers”. Pianta et al.’s “fast readers” experienced most of their growth from age 4.5 to 6 years, whereas our study begins at age six (first grade). Consistent with Pianta’s findings, the rapid catch-up readers grow fastest in the earliest years we observed. Our early bloomers (Class 1) have a mean trajectory that is approximately parallel to that of the steady progressors but simply starts higher, similar to the “fast reading development” class found by Kaplan (2002). Perhaps our early bloomers could be combined with our steady progressors to form a class similar to Pianta et al.’s “typical readers” because students in these classes all grow approximately linearly with class-specific mean curves that are approximately parallel, and the combined class would be only a little more heterogeneous than the early bloomers alone. Furthermore, the combined group includes 65% of the students, similar to the 70% found by Pianta et al. (2008) and the 72% found in the “normal development” group by Kaplan (2002). Unfortunately neither Kaplan (2002) nor Pianta et al. (2008) report within-class heterogeneity, making more formal comparison with their solutions difficult.

Other researchers have used the occasion numbers (0,1,2,3) as time variable for the NLSY (e.g., Bollen & Curran (2006, Chapter 2) and McNeish & Harring (2020)), and we therefore also present the results for this time scale in Appendix C of the Supplementary Material.

As mentioned above, we used priors for which estimation is stable so that we could introduce the application and model before discussing convergence issues. The rest of the article is structured as follows: We discuss Bayesian identification and estimation in Sections 3 and 4, respectively, before describing different kinds of problematic behavior and their diagnosis in Sections 5 and 6, respectively. Section 7 investigates strategies to detect and avoid the behaviors in a simulation study.

## 3 Bayesian identification

In Section 3.1, we define Bayesian identification in terms of likelihood identification based on a brief literature review. We then apply these concepts in Section 3.2 where we define specific types of nonidentifiability for finite mixture models, and Section 3.3 where we discuss consequences of these issues for estimation.

### 3.1 Bayesian versus likelihood identification

Loosely speaking, identification of parametric models concerns the existence of unique estimates of the model parameters by a given method (e.g., maximum likelihood estimation, or Bayesian estimation) for all possible data generated by the model.

Early work includes the expository article by Koopmans (1949) on identification of structural parameters in (non-Bayesian) linear simultaneous equation models. Interestingly, Koopmans & Reiersøl (1950) and Reiersøl (1950) also considered exploratory factor models, the latter in *Psychometrika*. The basic idea was that statistical inference regarding model parameters could be made in two steps: (1) inference from data to the reduced form parameters (e.g., variances and covariances) of the joint density of the data, and (2) inference from reduced form parameters to the structural parameters of the model representing the data-generating mechanism.

Inspired by this early work, Rothenberg (1971) and others have derived a number of identification results for general non-Bayesian parametric models. Let 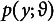 represent the likelihood of the data *y* in a model with fixed parameter vector 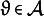 . Rothenberg provides several useful definitions for *likelihood* identifiability, including:

- Two parameter points 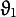 and 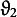 are *observationally equivalent* if 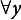 (where 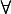 means “for all”): 

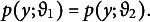

- A parameter point 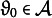 is *globally identified* if there is no other 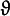 in the parameter space 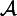 which is observationally equivalent. This form of identification is currently often called *point identification* (e.g., Lewbel, 2019).
- A parameter point 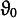 is *locally identified* if there exists an open neighbourhood of 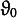 in which there is no other 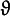 in 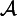 which is observationally equivalent. In this case, there may be several observationally equivalent parameter points but they are isolated from each other. See Bechger et al. (2001) on the distinction between global and local likelihood identifiability in a psychometric setting.
  For a given parameter point, local identification can in general be investigated by checking the rank of the information matrix at that point (Rothenberg, 1971). If applicable, local identification can alternatively be investigated by checking the rank of the Jacobian of the transformation from structural to reduced-form parameters (Wald, 1950).

The seminal work on *Bayesian* identification also occurred in the setting of linear simultaneous equation models, motivated by a desire to use priors to avoid imposing exact identifying restrictions (e.g., Drèze, 1974; Zellner, 1971). A general perspective on Bayesian identification problems was provided by Dawid (1979) in his investigation of the notion of conditional independence in statistics. Dawid lets Bayesian nonidentifiability of certain random parameters mean that the posterior of these parameters is the same as the prior, i.e., that the data provides no information on the parameters. His argument can be summarized as follows: Let 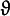 now represent a *random* parameter vector which can be decomposed into 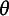 and 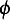 , a pair of (possibly vector-valued) parameters. Then 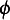 is not identified if 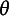 is a sufficient parameter in the sense that 
(2)

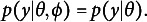

This is because, in this case, 
(3)

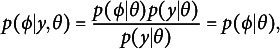

so that, “once we have learned about 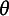 from the data, we learn nothing new about 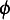 over and above what we knew already” (Dawid, 1979, p. 4). Because (2) implies that any two values of 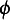 are observationally equivalent as defined by Rothenberg and others, this form of Bayesian nonidentifiability is a special case of classical likelihood nonidentifiability, where some parameters are redundant.

Poirier (1998) points out that it is useful to distinguish between *conditional uninformativeness*, defined in (3) and *marginal uninformativeness*, defined as 
(4)

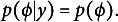

Marginal uninformativeness follows from conditional uninformativeness if and only if 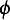 and 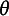 are a priori independent so that what we learn about 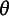 from the data does not provide information on 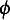 . Lack of independence can arise either from the conditional prior distribution 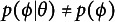 or from 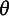 and 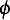 not being variation-free, i.e., their parameter space not being the product space. When 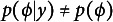 , there is Bayesian learning according to Kociecki (2013). Gustafson (2005) uses the term indirect learning when 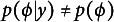 and learning about 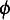 occurs only because of learning about 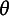 , as in (3).

As an example, Poirier (1998) considers a hierarchical model where 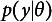 defines the likelihood in stage 1, 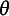 has a prior 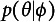 in stage 2, and the hyperparameter 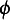 has a hyperprior 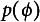 in stage 3, so that the joint posterior is 
(5)

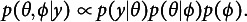

For example, in a variance-components model, 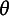 would include the vector of random intercepts and 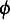 could be the random-intercept variance. Marginalizing over the “direct parameters” or latent variables 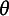 , the marginal posterior becomes 
(6)

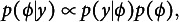

where 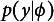 is the *marginal likelihood* used for likelihood inference in variance-components models (not the fully marginal likelihood as used in Bayes factors). Here the data are marginally informative about 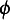 , but they add no information on 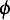 , given 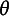 , because (2) holds. If the random intercepts were known, the variance 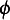 would be estimated by their sample variance, and the data would not provide further information.

In their discussion of model complexity, Spiegelhalter et al. (2002) distinguish between two ways of viewing a hierarchical model in terms of the parameters in focus. If 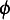 is in focus, the hierarchical model in (5) can be reduced to a non-hierarchical model as shown in (6), and if 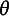 is in focus, it can be reduced to an alternative non-hierarchical model, 
(7)

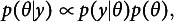

where 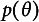 is the marginal prior, integrated over 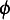 .

We argue that Bayesian identifiability of hierarchical models should be considered either for the parameters 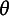 or 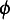 by integrating over the other parameters to obtain a non-hierarchical model. Such an approach would address Swartz et al. (2004)’s criticism of using likelihood identifiability as the definition for Bayesian identifiability. Swartz et al. (2004) present a hierarchical model for which 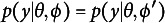 does not imply that 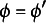 . Even though “there is no practical problem with this model” in that the marginal posterior means of 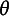 and 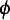 depend on the data, the model is not likelihood identified based on the likelihood 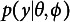 . This apparent contradiction is resolved by considering the marginal likelihood 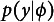 , a natural choice because likelihood inference for the model would be based on this likelihood (see also Appendix A).

Another reason to prefer the marginal likelihood is that it represents the sampling model or data-generating mechanism of interest in most situations. For example, in a variance-components model for clustered data, repeated samples would include new clusters, and the model is meant to have good predictive performance for out-of-sample clusters. Merkle et al. (2019) therefore argue that information criteria such as the Deviance Information Criterion (DIC, Spiegelhalter et al., 2002) and WAIC should be based on the marginal likelihood, and Gelman et al. (1996) refer to the corresponding predictive distribution as the *mixed predictive distribution*.

The important paper by Dawid (1979) discussed above considers a special case of Bayesian *non*-identifiability, where some parameters are redundant given the other parameters, but does not define identifiability. In contrast, Kociecki (2013) formally defines Bayesian identifiability, and we propose using the marginal likelihood in his definition when the model is hierarchical. Starting from the concept of identifiability of the sampling model ( 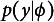 for hierarchical models) and viewing 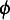 as random with support 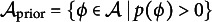 , Kociecki (2013) applies Bayes theorem, 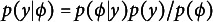 to define:

- A Bayesian model is globally identified at 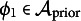 if and only if, 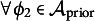 : 

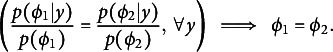

Likelihood identifiability and Bayesian identifiability are then equivalent if the prior has support on the full parameter space 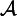 . However, when the support is restricted, 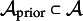 , there are instances where the Bayesian model may be identified even though the sampling model is not identified, for example if a prior restricts a parameter to be positive.

In the remainder of this article, we will define Bayesian identifiability as equivalent to marginal likelihood identifiability, and in the following subsections, we therefore focus on the latter, often referring to the non-Bayesian literature.

### 3.2 Identification in finite mixture models

Even if the models for the mixture components are globally identified, finite mixture models are not globally identified because for any parameter point 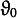 , all parameter points that correspond to permutations of the class labels are observationally equivalent, a phenomenon referred to as *labeling nonidentifiability* by Redner & Walker (1984). Since these observationally equivalent points are not in each other’s neighborhoods, permutation invariance does not violate local identification. We assume that permutation invariance has been taken care of, for instance by labeling the classes in order of increasing class size. *Generic nonidentifiability* of finite mixture models refers to the existence of several observationally equivalent parameter points that do not correspond to different permutations of class labels (see Frühwirth-Schnatter, 2006, Section 1.3.4).

Lewbel (2019) requires local or global “point” identification to hold at all possible parameter points 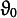 because it should hold at the true parameter values which are unknown to us. Finite mixture models are then only *set identified* because point identification does not hold everywhere—at some parameter points there is a set of parameters that are observationally equivalent. In our setting, for a parameter point 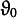 with one or more class probabilities equal to zero, all parameters that differ from 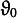 only in terms of the class-specific parameters for the classes with zero probability are observationally equivalent to 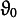 —they are in what Lewbel (2019) calls the “identified set.” Similarly, when the parameters of two (or more) classes are identical, all parameter points for which the class-probabilities for the indistinguishable classes have the same sum are in the identified set. Both types of parameter points can be referred to as degenerate because the model reduces to a mixture with fewer mixture components. We therefore say that finite mixture models are not locally identified at degenerate parameter points.

The idea of parameters being locally nonidentified in *parts* of the parameter space is discussed by Andrews & Cheng (2012). They consider the situation where the parameter vector can be written as ( 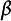 , 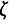 , 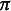 ) and 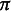 is identified if and only if 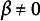 , whereas 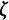 is not related to the identification of 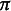 and ( 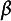 , 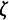 ) are always identified. When 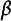 is close to zero, 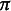 is *weakly* identified. A finite mixture model is clearly an example of this situation with the smallest class probability, 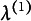 , corresponding to 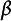 , the class-specific parameters for Class 1 corresponding to 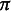 and the remaining parameters to 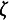 . Another example is a confirmatory factor model with two common factors, each measured by two variables. Here, the model is identified except at parameter points with zero covariance between the factors (e.g., Kenny, 1979, p. 178; Skrondal & Rabe-Hesketh, 2004, pp. 148–149).

Another problem with a degenerate parameter point 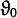 is that there is at least one other degenerate parameter point 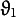 in a different part of the parameter space that is observationally equivalent, and this is another violation of global identification, similar to labeling nonidentifiability. We will call this kind of violation *degenerate nonidentifiability*, a term used by Kim & Lindsay (2015). This notion was previously discussed in the context of specifying more than the actual number of classes (e.g., Crawford, 1994; Rousseau & Mengersen, 2011) and referred to as “nonidentifiability due to overfitting” by Crawford (1994). An example of degenerate nonidentifiability given by Kim & Lindsay (2015, p. 748) and Lindsay (1995, p. 74) is a two-component mixture model that *degenerates* to a one-component mixture if one of the class probabilities is zero or if the component-specific parameters are equal. Hence, a one-component mixture cannot be distinguished from these different versions of a two-component mixture. For a three-component model, there are more than two ways of degenerating to a two-class model, namely one class probability is zero, the first (e.g., smaller) class of the two-class model is represented by two classes with equal class-specific parameters or the second (e.g., larger) class of the two-class model is represented by two classes with equal class-specific parameters.

### 3.3 Degenerate estimates and degenerate draws from the posterior

The classical treatment of identification in parametric models concerns the identification at true but unknown parameter points. However, work has also been concerned with identification at the parameter *estimate*, called *empirical* identification (see Kenny (1979, pp. 49–50) and Rindskopf (1984)). In structural equation models, empirical local identification has been assessed by checking the rank of the estimated information matrix at the maximum likelihood estimates (McDonald & Krane, 1977; Wiley, 1973). In latent class models, Goodman (1984) discusses local identification and local empirical identification at the maximum likelihood estimates by investigating the rank of the Jacobian of the transformation from the model parameters to the cell probabilities of the contingency table (i.e., the reduced form parameters).

Somewhat related but different from empirical identification, we will be concerned with identification at *draws from the posterior* during MCMC estimation. Drawing degenerate parameter vectors can lead to estimation problems. For example, when a draw of 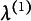 is zero or close to zero, the Class-1-specific parameters are only weakly identified in that region of the parameter space and will be approximately drawn from their priors. Draws of Class-1-specific parameters can then be incompatible with the data so that, locally, the posterior will strongly favor 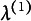 equal to or close to zero. This way, miniscule-class sampling becomes self-perpetuating. We refer to this phenomenon as “self-perpetuating sampling of near-degenerate parameters.”

A different problem described by Gelman et al. (2014, p. 393) can also be viewed as self-perpetuating sampling of near-degenerate parameters. They consider a hierarchical model (equivalent to a *t*-distribution) where the variances 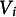 of the units have a scaled inv- 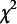 distribution with scale parameter 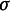 . Parameter draws with 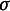 close to 0 are near-degenerate because the model degenerates to a non-hierarchical model when 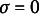 . For such draws, Gelman et al. (2014, p. 295) write that “the conditional distribution [of 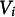 given 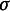 ] will then cause the 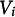 ’s to be sampled with values near zero, and then the conditional distribution of 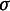 will be near zero, and so on.” In other words, the behavior is self-perpetuating.

There does not appear to be a similar mechanism that would cause the other kind of degeneracy in finite mixture models to be self-perpetuating, where two or more “twinlike” classes have (nearly) identical class-specific parameters. However, when a model with *K* classes is specified and a degenerate version equivalent to a model with 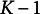 (or fewer) classes corresponds to a mode of the posterior, twinlike behavior may persist for many iterations. In this case, several different degeneracies can be (nearly) observationally equivalent, each corresponding to a different posterior mode. It is then possible that one chain remains near one of the modes, for instance, with two classes having (nearly) identical parameter values, while another chain remains near another mode (that is nearly observationally equivalent), with one class probability (nearly) equal to zero. If such behavior occurs, posterior means of parameters across chains will no longer be meaningful, unless appropriate reparameterization is performed before averaging. This phenomenon, due to degenerate nonidentifiability, is similar to the label switching problem, due to labeling nonidentifiability. These types of nonidentifiability are generally not a problem for maximum likelihood estimation which tends to converge to one of several observationally equivalent parameter points as long as they are in different parts of the parameter space.

## 4 Bayesian estimation in Stan

### 4.1 Brief overview of HMC

In Markov chain Monte Carlo (MCMC), new parameter values 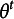 are sampled in iteration *t* conditional on current parameter values 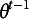 in such a way that (1) the time series of parameter values becomes stationary after a “burn-in” or “warmup” period, and (2) the stationary distribution is the required posterior distribution, 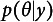 . To achieve (2), the transition probabilities are such that, if the marginal posterior probability density of 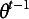 (given *y*) is the target distribution, then the marginal density does not change and therefore the marginal density of 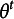 is also the target density.

Metropolis–Hastings algorithms proceed by generating a proposal 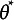 (“Proposal Step”) and deciding whether to accept the proposal, i.e., whether to set 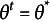 , according to a rule that ensures convergence of the distribution to the target distribution (“Acceptance Step”). In HMC (e.g., Betancourt, 2018; Neal, 2011), the proposals 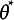 are generated using Hamiltonian dynamics, briefly described below, with the advantage over traditional methods, such as Metropolis and Gibbs sampling (implemented in OpenBUGS, JAGS, and Mplus), that the parameter space tends to be sampled more efficiently, in terms of the number of iterations (e.g., Betancourt & Girolami, 2015). We give a brief overview of HMC, synthesizing descriptions by Betancourt & Girolami (2015), Gelman et al. (2014), and the Stan Reference Manual (Stan Development Team, 2024).

#### 4.1.1 Proposal Step

To generate the proposal 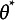 for iteration *t*, the current parameter values 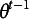 are treated as the initial coordinates of a particle whose movements are governed by Hamiltonian dynamics, a system of differential equations whose solution describes motion, for instance of a particle in a physical system. To simplify notation, we will use 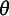 without any superscript to denote the coordinates along the trajectory that are initially set to 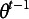 and whose value after some amount of time is used as the proposal 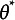 .

To visualize the dynamics in three dimensions, we assume that the two-dimensional parameter vector 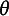 represents coordinates on a horizontal plane, along the left-to-right and back-to-front dimensions. A possibly misshapen bowl situated above this plane contains a particle whose third coordinate, its height, is determined by the surface of the bowl. This height is given by 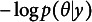 , where 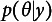 is the posterior probability density function of the parameters 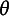 given the data *y*, which is the target distribution we aim to sample from. This height represents the potential energy due to gravity, i.e., the amount of energy needed to lift the particle to that height (potential energy would actually be height times mass times acceleration due to gravity). The basic idea here is that the particle will tend to fall “down” into regions of the bowl where the posterior density is greater.

The reason the particle does not fall to the bottom and stay there, which would mean that the full parameter space would not get explored, is that it also has speed and *momentum*. Specifically, for each model parameter (or particle coordinate on the horizontal plane), there is an auxiliary random variable that represents the momentum of the particle in the corresponding direction. The initial value of the momentum vector, denoted 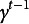 , is sampled from a multivariate normal distribution with zero means and covariance matrix *M*, and the corresponding density is denoted 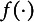 . As the location changes, so does the momentum, and we use 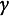 without a superscript to denote the evolving momentum. Due to its momentum, the particle has kinetic energy given by 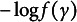 . According to the laws of physics for frictionless motion, the particle follows a deterministic trajectory that conserves the total amount of energy (potential plus kinetic), 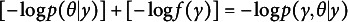 and hence keeps the joint posterior density 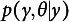 constant. As the particle loses height, it loses potential energy 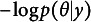 and correspondingly gains kinetic energy 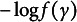 and hence gains speed and momentum, allowing it to gain height again (if the surface of the bowl rises in the direction of its motion) at the expense of losing momentum. You may find it useful to play with the following animation created by Chi Feng to get a better understanding of the dynamics (as well as the no-U-turn idea described below): https://chi-feng.github.io/mcmc-demo/app.html.

In order to compute the trajectory of the particle based on differential equations (the Hamiltonian equations), the position and momentum vector of the particle are updated at time-intervals, or “steps” of size 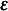 . Specifically, the increase in momentum depends on the decrease in potential energy along the trajectory, and this is approximated by treating the downward slope of the bowl (i.e., the gradient of the log posterior) as constant along the path for duration 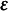 . The momentum after half the time interval 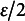 is computed as 

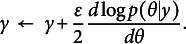

Having updated the momentum, it is treated as constant from the beginning to the end of the interval to compute the next location (i.e., the parameter values) along the path. After a *full* time interval 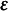 , the location becomes 

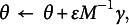

where 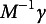 is the velocity (rate of change in position per time interval, or derivative of position with respect to time) and *M* is sometimes called a Euclidean metric or a mass matrix, the latter because momentum equals mass times velocity. The momentum at this full-step is then updated before proceeding with the next time interval. This method for computing the dynamics is called a “leapfrog integrator.”

The smaller 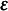 , the more accurate the trajectory at the cost of increased computation time. Steps that are too large can result in errors due to assuming that 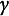 is constant and that the height 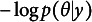 changes linearly during the time interval 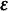 . These errors can be quantified by comparing the total energy at a point on the trajectory with the initial energy. When the difference becomes pronounced, a “divergent transition” is said to occur.

#### 4.1.2 Acceptance Step

The location and momentum vectors after *L* time intervals become the proposals, denoted 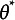 and 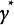 . Then 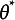 is accepted with probability 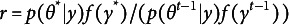 if 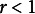 and with probability 1 otherwise. Note that 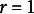 if the total energy is conserved, i.e., the marginal posterior distribution does not change. This will happen when there are no errors of approximation to the true trajectory or when 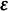 approaches 0. If 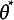 is not accepted, the next 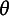 is 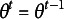 . For the next iteration, a new momentum vector is drawn, leading to a new proposal as described in the proposal step above.

### 4.2 No U-Turn Sampler (NUTS) and HMC parameters

If the “integration time” 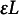 is too small, the chain moves too slowly through the parameter space, and if it is too large, the particle may “loop back and retrace its steps” (Hoffman & Gelman, 2014). Hoffman and Gelman therefore proposed NUTS. Briefly, the simplest form of NUTS involves building a tree of evaluations of the trajectory. At treedepth (or tree height) *j* (starting with 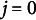 ), the dynamics are computed forward in time or backward in time (direction chosen randomly) for 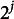 leapfrog steps until the next step would result in a decrease in the distance between the position of the particle and its starting point, called a “U-turn.” Then 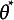 and 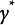 are sampled from among all points computed.

During warmup, the mass matrix *M* and step size 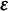 are adapted (see Stan Development Team, 2024, for details). Essentially, *M* is an estimate of the posterior covariance matrix of 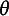 , and the step size is adapted to achieve a target Metropolis rejection rate denoted delta in Stan, with default value 0.8. Users have the option to define the initial step size for the HMC sampler, which acts as a starting point for adapting the step size and is not necessarily the one used in the first warmup iteration (Zhang, 2020).

Users can set the target acceptance rate delta and a value greater than the default of 0.8 will result in smaller step sizes. While this enhances the effective sample size, it may also extend the time required per iteration (Stan Development Team, 2024). Additionally, users can specify the maximum treedepth for NUTS, which defaults to 10 in RStan (Stan Development Team, 2023).

### 4.3 Known challenges for HMC and Stan diagnostics

A challenge with any MCMC algorithm is ensuring that the stationary distribution was reached within the designated warmup period. A useful diagnostic procedure is to start several Markov chains with different starting values (e.g., randomly drawn). Once all these chains have reached the same stationary distribution, they should have the same properties and should “mix” in the sense that the traceplots occupy the same region. Then the total variance across the chains (the within-chain variance plus the between-chain variance) should not be greater than the pooled within-chain variance. The traditional 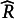 diagnostic (Gelman & Rubin, 1992) is therefore roughly the total variance divided by the within-chain variance. 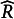 should be close to 1 and a value greater than 1.10 for any of the parameters is often considered a sign that the stationary distribution has not been reached. Vehtari et al. (2021) proposed using the maximum of the rank-normalized split- 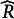 and the rank-normalized folded-split- 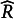 , which we use here and simply denote as 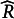 for short. Briefly, the rank-normalized split- 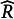 is obtained by rank-normalizing the parameter draws so that they approximately follow a standard normal distribution and treating the first and second half of each chain as separate chains before applying the traditional formula for 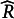 . The rank-normalized folded-split- 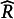 is obtained by transforming parameter draws to their absolute deviation from the median and then applying the computations for the rank-normalized split- 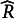 .

Another issue with any MCMC algorithm is that the parameter draws are not independent so that the number of iterations cannot be viewed as the sample size when estimating Monte Carlo errors for estimates of the posterior means. The effective sample size (ESS) can be estimated (see, e.g., Gelman et al., 2014, Section 11.5). When the ESS is much smaller than the number of iterations, this signals that the chain is moving very slowly through the parameter space and may be encountering problems.

As mentioned in Section 4.1, a divergent transition is said to occur in HMC when the Hamiltonian (sum of kinetic and potential energy) at a point along the computed trajectory becomes too different from the initial Hamiltonian, implying that the computed trajectory has diverged from the true trajectory. The Stan Reference Manual (Section 15.5) explains that “positions along the simulated trajectory after the Hamiltonian diverges will never be selected as the next draw of the MCMC algorithm, potentially reducing HMC to a simple random walk and biasing estimates by not being able to thoroughly explore the posterior distribution.” Divergent transitions (or divergent iterations) can occur when 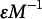 is too large in some direction(s) for the linear approximations of the leapfrog integrator to hold, due to what Betancourt (2020) describes as “neighborhoods of high curvature.” Stan flags iterations for which divergent transitions occurred.

The maximum treedepth is reached when no U-turn is encountered after computing 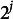 leapfrog steps for each treedepth *j* from 0 to the maximum. According to the Stan Reference Manual (Stan Development Team, 2024), a treedepth equal to the maximum may be a sign of poor adaptation of the tuning parameters (e.g., mass matrix *M* and step size 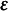 ), may be due to targeting a very high acceptance rate delta, or may indicate a difficult posterior from which to sample.

The Stan Reference Manual states (Section 15.5): “The primary cause of divergent transitions …is highly varying posterior curvature, for which small step sizes are too inefficient in some regions and diverge in other regions. If the step size is too small, the sampler becomes inefficient and halts before making a U-turn (hits the maximum treedepth in NUTS); if the step size is too large, the Hamiltonian simulation diverges.”

Betancourt (2016) introduced the estimated Bayesian fraction of missing information (E-BFMI) to assess the efficiency of the momentum resampling at the beginning of each HMC iteration. The E-BFMI is the ratio of the variance of *changes* in the Hamiltonian between adjacent MCMC iterations (due to the kinetic energy changing when the momentum is resampled) to the variance of the Hamiltonian itself (due to changes in both kinetic and potential energy). Betancourt (2018) states that, empirically, values below 0.3 have been proven problematic.

### 4.4 Growth mixture modeling

#### 4.4.1 Implementation in Stan

When finite mixture models are estimated by MCMC, it is common to sample the discrete latent variable 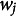 that represents class membership along with the model parameters and any continuous random effects or latent variables. However, HMC works only with continuous parameters and the likelihood must therefore be specified marginal over the discrete latent variable. Demonstrations of how to specify such marginal likelihoods in Stan can be found in Betancourt (2017), the finite mixture model example in the Stan User’s Guide (Stan Development Team, 2021), and the tutorial by Ji et al. (2021), as well as Appendix B.

We also marginalize over the random intercept and slope instead of treating these random effects as model parameters. This approach, although unconventional, is also employed in blavaan (Merkle & Rosseel, 2018; Merkle et al., 2021). One reason is potential computational efficiency gains due to sampling far fewer parameters (Merkle et al., 2021). Another reason is that model assessment based on predictive distributions is more meaningful if *mixed* posterior predictive distributions, based on marginal likelihoods, are used (Merkle et al., 2019).

As shown in (1), the class-specific models for our GMM are linear mixed models, with a random intercept and random slope of time. Therefore, for a given class *k*, the marginal joint density of the responses 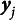 for a subject is multivariate normal with means 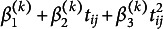 and covariance matrix 
(8)

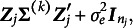

where 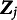 is the design matrix for the random effects with a column of 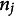 ones for the random intercept and a column of time-points 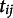 , 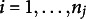 , for the random slopes, and 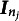 is a 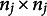 identity matrix. This multivariate density can be evaluated directly as 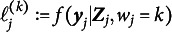 instead of conditioning on the random effects as is typically done in MCMC estimation of (generalized) linear mixed models. The likelihood for the entire dataset then becomes 
(9)

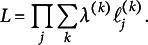



The priors for the model parameters are as discussed in Section 2.3.1. We used random starting values for these parameters generated by Stan. Specifically, draws are obtained from Uniform(-2,2) distributions and, if needed, transformations are applied to satisfy parameter bounds, such as the exponential to obtain non-negative standard deviations and the softmax function to obtain probabilities on a simplex.

The complete Stan code for estimating our GMM is included in Appendix B.

#### 4.4.2 Addressing label switching by relabeling

Label switching occurs because the likelihood and posterior are invariant to permutations of the latent class labels as mentioned in Section 3.2. Latent class labels can differ between chains for any MCMC algorithm that uses random starting values. Within-chain label switching can also occur. Interestingly, this is unlikely when the class labels 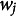 associated with the units *j* are sampled along with the model parameters as in Gibbs sampling. All these labels are unlikely to switch simultaneously from one iteration to the next (e.g., Celeux et al., 2000; Kamary, 2016; Lee et al., 2009). When between-chain or within-chain label switching occurs, posterior means of class-specific parameters are no longer meaningful.

A common solution to the problem is to impose an order constraint on one of the class-specific parameters, such as the mean intercept 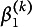 , so that the posterior is no longer invariant to label permutations. However, the approach relies on finding a parameter that takes on sufficiently different values across classes and is often ineffective (e.g., Celeux et al., 2000; Marin & Robert, 2007, pp. 163–164; Stephens, 2000).

In Mplus, label switching between chains is addressed by introducing a preliminary stage of 50 iterations (by default) during which all chains are identical so that the chains are likely to have a common labeling in the second stage (Asparouhov & Muthén, 2023). This approach does not protect against within-chain label switching and could undermine 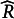 as a convergence diagnostics because this statistic is based on independent chains with different starting values. Asparouhov & Muthén (2023) also point out that the joint posterior mode can be used instead of posterior means. However, interval estimation is then precluded and the point estimates are likely to be unstable (e.g., Celeux et al., 2006).

Here we do not use any of the strategies discussed above. Instead, we relabel the parameters after MCMC sampling is complete using the Kullback–Leibler algorithm (or “algorithm 2”) developed by Stephens (2000). Loosely, this algorithm finds permutations of the labels for each iteration to make the corresponding posterior classification probabilities of the *n* individuals across the *K* classes as similar as possible across iterations. The algorithm is available in the label.switching package (Papastamoulis, 2016) in R. This package implements various relabeling algorithms, provides post-relabeling convergence diagnostics, such as 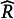 , and provides a measure of similarity between label permutations from different relabeling algorithms. Ji et al. (2021) offer both the rationale and code for addressing label switching in latent class models using the label.switching package. The GitHub given in the Introduction includes the PP_sss function for extracting the original posterior draws from a GMM Stan object and restructuring these draws so they can be passed to the post_processing function for relabeling.

## 5 Problematic estimation behavior

The analysis of the NLSY data reported in Section 2.4 showed no problematic behavior when using D10N50 priors. In this section, we alter the priors and show some of the problematic behavior that can occur. We will use the same model as described in Section 2.3.1 but with 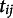 equal to the occasion number (0,1,2,3) instead of age because researchers employing software for balanced longitudinal data can then compare their results with ours.

Appendix C of the Supplementary Material reports estimates for this time variable based on D10N50 priors. To assess potential shrinkage toward equal probabilities of 1/3 due to the Dirichlet prior with 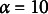 , we also obtained maximum likelihood estimates from the flexmix package (version 2.3-19, Grün & Leisch, 2008, 2023) in R. The posterior mean estimates are 0.267, 0.286, and 0.447, and they are closer to 0.333 than the maximum likelihood estimates of 0.214, 0.213, and 0.574, but the latter fell within the 90% credible intervals of the former (not shown). No local maxima were found after running flexmix with 1,500 different sets of starting values, suggesting that the likelihood is unimodal (apart from the label permutation invariance).

In this section, we specify 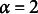 and 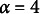 as vague/diffuse and mildly informative options for the Dirichlet distribution and 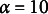 to moderately favor equal class probabilities (denoted D2, D4, and D10, respectively). For the intercept and slope standard deviation parameters, we specify half-normal priors with scale parameter 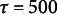 and 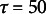 (denoted N500 and N50, respectively) and half-Cauchy priors with scale parameter 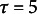 (denoted C5).

For some combinations of these priors (D2C5, D4N100, and D2N500) and the three-class GMM, we ran twenty chains with 1,000 warmup iterations and 1,000 post-warmup iterations for each set of priors. After relabeling as described in Section 4.4.2, we inspected traceplots and other summaries of the results to find evidence of problematic behavior.

**Figure 2:**
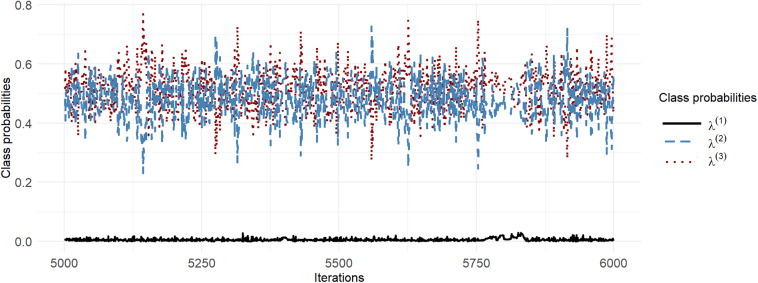
(Color online) Traceplot for Chain 6 for D2N500 priors where the Class-1 probability 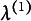 is persistently close to 0.

**Figure 3:**
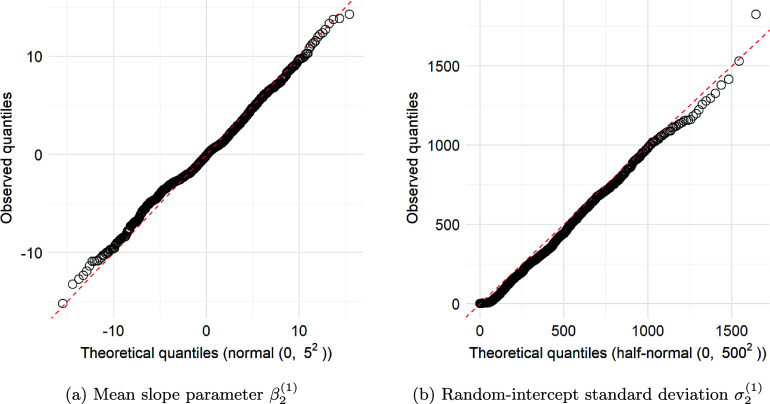
Q-Q plots of mean slope and random-intercept standard deviation parameter draws for Class 1 compared with their priors for Chain 6 with D2N500 priors.

Figure 2 shows a traceplot for the class probabilities for Chain 6 with D2N500 priors, where the draws of the Class-1 probability, 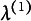 , are persistently close to 0. As expected because the Class-1 parameters are only weakly identified when 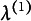 is close to 0, we found that these parameters were approximately sampled from their *prior* distributions (see Figure 3 for examples). These samples are not likely to be compatible with the data, so that 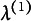 remains close to zero for all 1,000 iterations.

**Figure 4:**
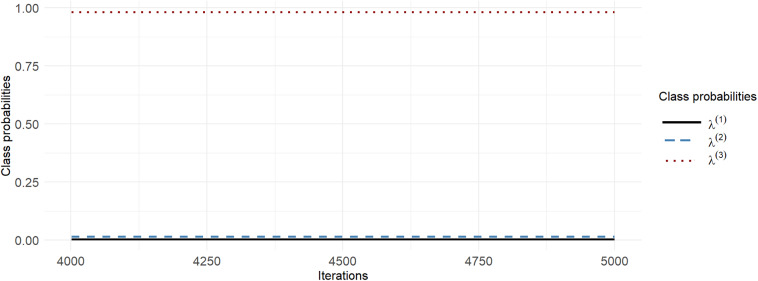
(Color online) Traceplot for Chain 5 with D2C5 priors where 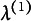 and 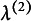 are close to 0 and the chain is completely stuck.

**Figure 5:**
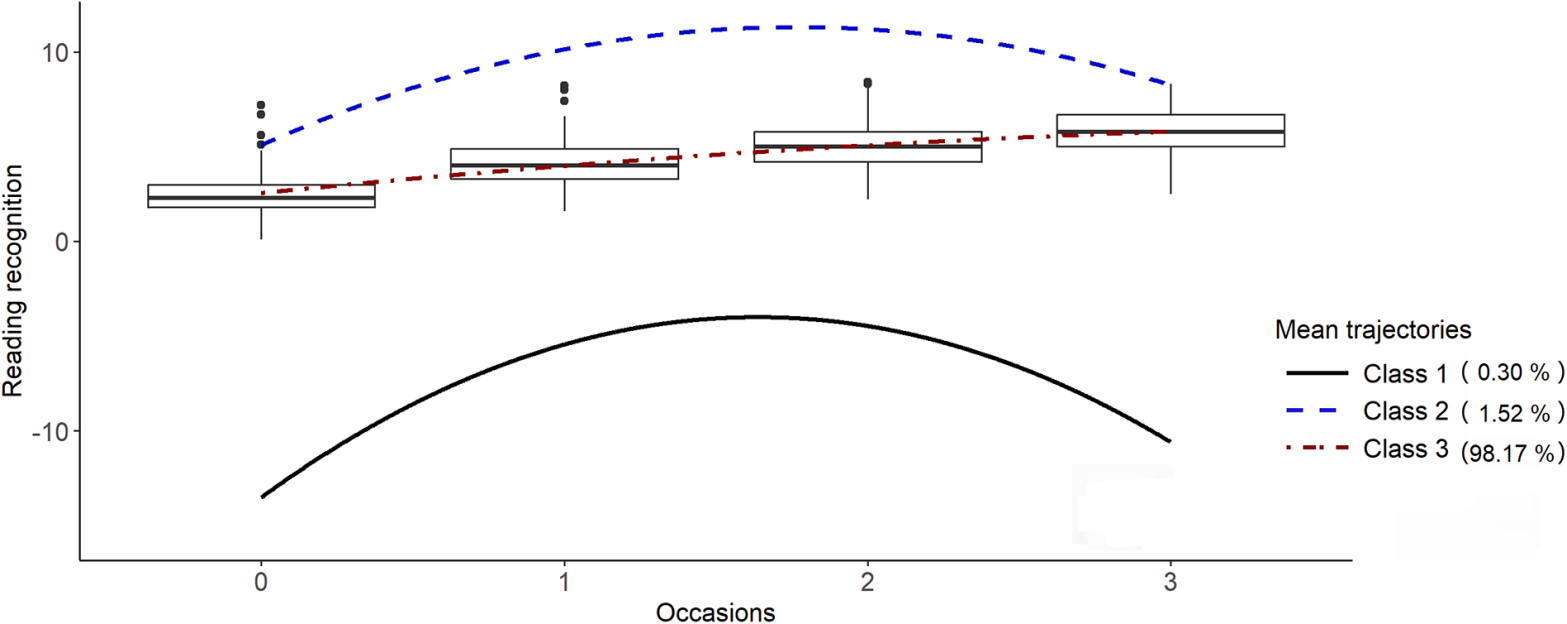
(Color online) Class-specific mean trajectories for Chain 5 for the D2C5 priors and box-plots of reading scores.

Chain 5 with the D2C5 priors exhibits even more problematic behavior as shown in the traceplot in Figure 4. Here, 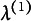 and 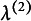 are both close to zero, and the chain is completely stuck. As shown in Figure 5, the mean trajectories for Classes 1 and 2 (implied by the posterior draws of the corresponding parameters in Chain 5) are mostly outside the range of the data, forcing the probability parameters to remain close to zero.

We have therefore found evidence for self-perpetuating sampling of near-degenerate parameters that can manifest in two ways. We will use the term “miniscule-class” behavior to denote when the probability parameter(s) of one or more classes fluctuate around a near-zero value with a small standard deviation and the terms “stuck-sequence” and “stuck-chain” behavior when the chain is completely stuck for a sequence of consecutive iterations or remains stuck for the entire chain with one or more class-probabilities near zero.

We were not able to find highly problematic behavior due to class-specific parameters being equal, or “twinlike-class behavior,” either for the NLSY data or for the simulations reported in Section 7.2. Appendix D of the Supplementary Material shows an example with D4N100 priors where the posterior means of the class-specific parameters for Classes 1 and 2 are very similar. However, these parameters were never extremely close in a given iteration. Apart from slightly smaller ESS compared with a chain for D10N50 where the posterior means were less similar, there were no signs of problematic behavior.

## 6 Diagnostics for problematic behavior

In Section 6.1 we introduce some new diagnostics designed specifically for detecting the problematic behavior discussed in this article and recommend steps for diagnosing these behaviors. The next subsection applies the diagnostics to the NLSY data and presents results. All functions referred to here can be found in the GitHub given in the Introduction.

### 6.1 Procedure

- **Step 1:** *Initial Screening based on* 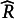

  Begin by examining whether any parameter exceeds the commonly recommended threshold of 1.10 for the 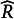 statistic. If such instances are identified, record the number of parameters exceeding this threshold and calculate the mean 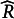 for all relevant parameters.
  However, relying solely on 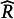 may be insufficient. Even if 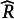 does not exceed 1.10, proceed to Steps 2–4 to detect “stuck-sequence” (Step 2), “twinlike-class” (Step 3), and “miniscule-class” (Step 4) behavior discussed in Section 5.
- **Step 2:** *Stuck-sequence diagnostic*
  To detect when parameter draws do not change for several consecutive iterations, we suggest computing the moving standard deviation for one of the parameters, such as the smallest class probability parameter 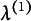 . For a window size of 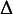 , the moving standard deviation of 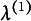 at iteration 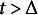 is defined as 

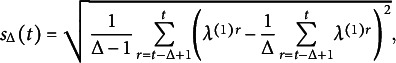

where 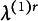 is the draw of 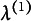 in iteration *r*. If the moving standard deviation remains at zero for *q* consecutive windows, this means that the chain is stuck for *q*+ 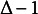 consecutive iterations. We suggest recording the number of such stuck sequences and the lengths of these sequences.
  We provide the stuck_by_chain function that allows users to customize the window size for the moving standard deviation and the minimum length that defines a stuck sequence. This function informs users about which chains exhibit stuck sequences, how many chains are affected, where these sequences are detected, the lengths of the stuck sequences, and which chains are stuck persistently.
  Longer stuck sequences can also be detected in traceplots. In addition, we expect a high 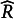 in situations where there are minimal changes within a chain or if a chain persistently remains stuck. This is because the contribution of such a chain to the within-chain variance in the denominator approaches zero. The limited number of distinct draws will also be reflected in a small ESS.
- **Step 3:** *Twinlike-class diagnostic*
  To detect when class-specific parameters for a pair of classes are nearly indistinguishable, we propose a distinguishability index (DI) that can be calculated for each iteration of the chain.
  If the parameters for classes *k* and *l* are identical, then the class-specific joint densities of the responses for any subject *j* should be the same for both classes, i.e., 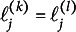 , where 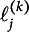 is defined in Section 4.4.1. If, additionally, 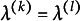 , the posterior probabilities of belonging to classes *k* and *l* will be identical for all subjects. Our index therefore starts with the conditional posterior probability of belonging to class *k*, given that the individual belongs either to class *k* or *l* and under “prior ignorance” 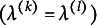 , 

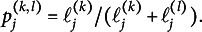

  To summarize how close to 0.5 these probabilities are across subjects, we use the corresponding average conditional entropy, defined as 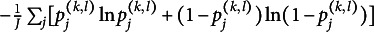 , which takes the value 0 if 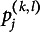 equals 0 or 1 (no classification uncertainty) and the value 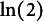 if 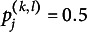 , corresponding to 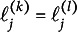 for all subjects *j* (greatest classification uncertainty). We then define the DI as 

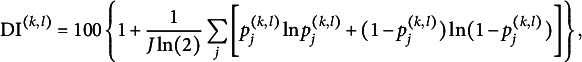

which takes the value 0 if 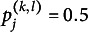 for all *j* (indistinguishable classes) and the value 100 if 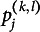 equals 0 or 1 for all *j* (most distinguishable classes).
  We provide the DI function to calculate DI and the twinlike_classes function to produce traceplots of class probabilities for all classes and DI plots for all class pairs, aligned vertically for comparison. This function also returns detailed information about chains where the DI is below a certain threshold, indicating twinlike behavior.
- **Step 4:** *Miniscule-class diagnostic*
  To identify sequences of iterations in which the smallest class probability fluctuates (with small variance) near zero, we suggest inspecting the traceplot of the smallest class probability, 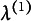 , along with its moving average and moving standard deviation.
  In instances where the chain alternates between normal behavior and fluctuating-near-zero behavior, we can apply a clustering algorithm, such as K-means, to the moving averages and moving standard deviations. If the smaller centroid (both for the moving average and moving standard deviation) is close to (0,0), iterations in that class exhibit miniscule-class behavior. To be conservative in the classification, the iterations with moving average values above a given percentile (such as the top 10%) in that class can be removed. We suggest reporting the frequency of sequences of consecutive iterations with miniscule-class behavior and the durations of these sequences.
  Additionally, when miniscule-class behavior occurs for Class 1, the Class-1-specific parameters are approximately drawn from their priors and are not likely to be compatible with the data. Consequently, the posterior probability of belonging to Class 1 will be close to zero for most subjects and hence the DI, defined in Step 3, will be close to 100 when comparing Class 1 with any of the other classes. We propose using the DI as another diagnostic for minuscule-class behavior.
  Our diagnostics_graphs function can be used to assess miniscule-class behavior visually, with customization options for users’ needs. This function produces traceplots, moving average and standard deviation plots, and DI plots, aligning them vertically. Additionally, the function offers detailed warnings and information regarding the chains where miniscule-class behavior is observed.

### 6.2 Using the diagnostics

The diagnostic process is illustrated by applying it to sets of 20 chains of 1,000 post-warmup MCMC draws, for the NLSY data. We use different combinations of priors (D2C5, D2N500, D4N100, and D6N500) for which we expect problematic behavior to occur. No evidence of twin-like behavior was found, but we illustrate the other types of problematic behavior below.

#### 6.2.1 Persistently stuck chains

**Figure 6:**
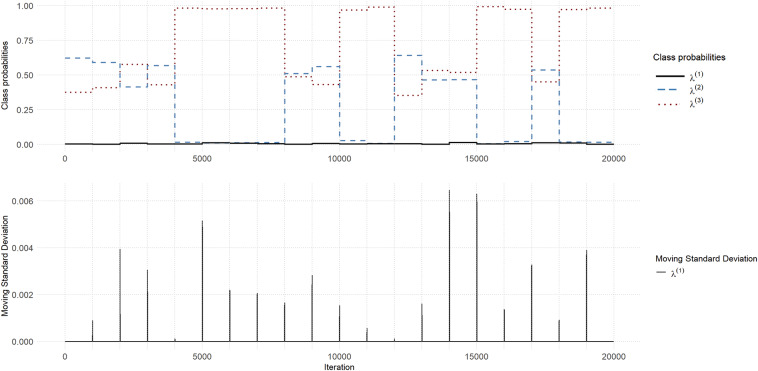
(Color online) Persistently stuck chains for D2C5 priors. *Note*: Top Panel: Traceplot of 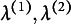 , and 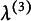 Bottom Panel: Moving standard deviation of 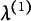 .

With D2C5 priors, the moving standard deviation of 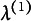 , with a window size of 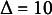 remains at zero for each entire chain (and changes by a tiny amount between chains), implying that the chains are persistently stuck, as shown in Figure 6.

When employing the summarise_draws function in the posterior package (version 1.5.0, Bürkner et al., 2023; Vehtari et al., 2021) in R, the 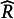 values are flagged as Inf (infinite) in this case. This is due to the within-chain variance being 0 because all chains are stuck. However, the monitor function in Stan reports an 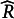 value of 1 when all chains are persistently stuck, which is misleading. Regarding the ESS, the summarise_draws and monitor functions in Stan both provide two estimators, the bulk-ESS and the tail-ESS. Whereas the summarise_draws function reports NA for both, which indicates that the draws are constant, the monitor function in Stan incorrectly reports 1,000 for both, which is misleading.

#### 6.2.2 Miniscule-class sequences

**Figure 7:**
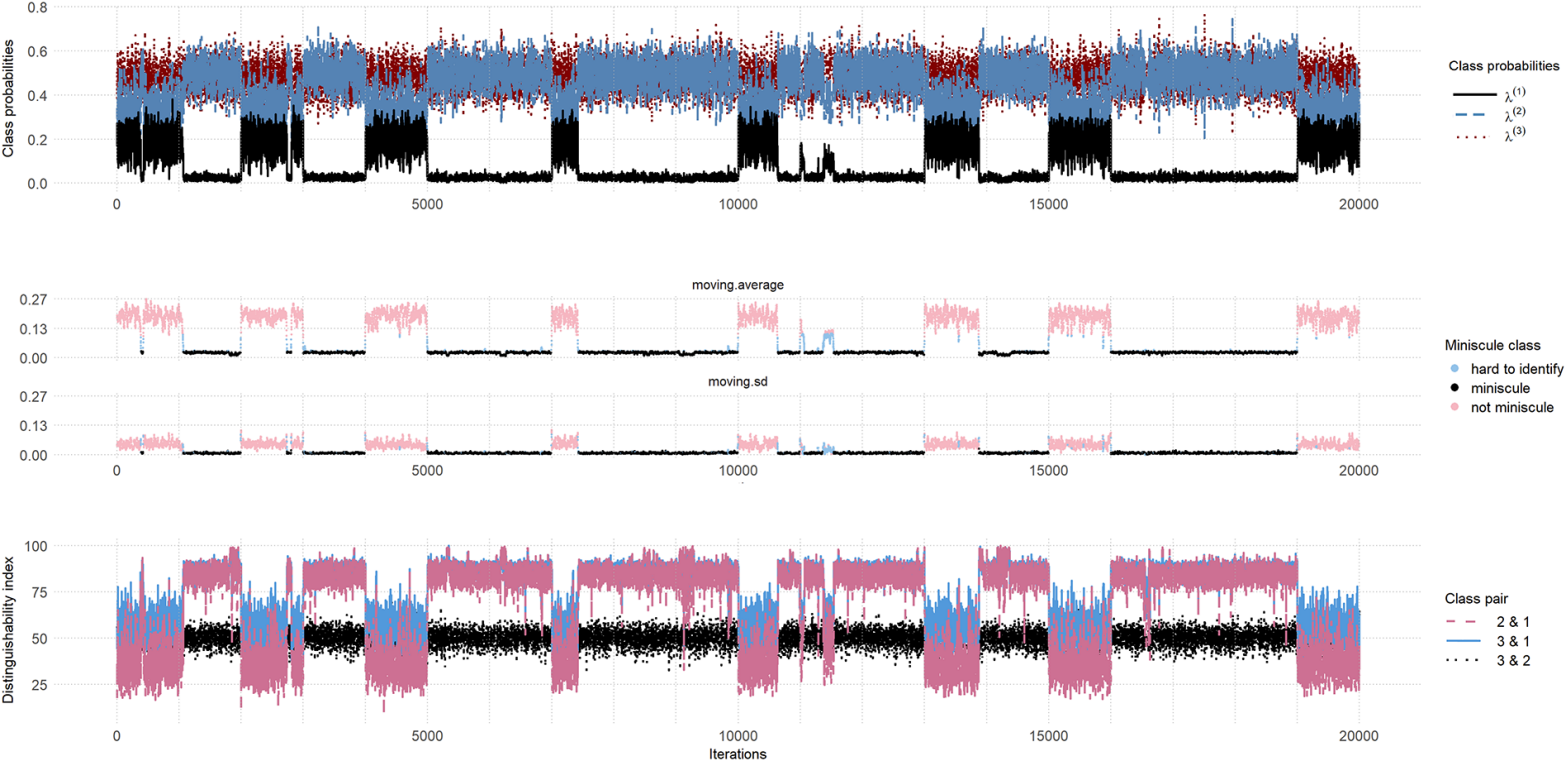
(Color online) Miniscule-class behavior for D4N100 priors. *Note*: Top Panel: Traceplot of 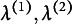 , and 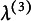 Mid Panel: Moving average and moving standard deviation of 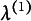 Bottom Panel: Distinguishability index for all class pairs.

The top panel of Figure 7 is a traceplot of the latent class probabilities for all 20 chains for the D4N100 priors. Out of the 21 parameters, ten have 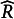 values exceeding 1.10 and the mean 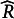 for all parameters is 1.182. The stuck-sequence diagnostic identifies one stuck sequence of length 11 at iteration 16,554 (chain 17).

The middle panel in the figure shows the moving averages and moving standard deviations, color-coded according to the classification of the K-means algorithm, where black represents miniscule-class sequences, pink is normal behavior, and blue means not classified (largest 10% of values classified as miniscule by K-means algorithm). In total, 11,761 out of 20,000 iterations are classified as minuscule class behavior. The DI-plot in the bottom panel of Figure 7 supports this miniscule-class behavior. Specifically, when 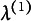 is near zero, 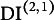 and 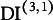 tend to be extremely large, typically exceeding 75 and often approaching 100. This is expected as the Class-1 trajectory parameters are drawn approximately from their priors and are therefore not likely to be compatible with the data, leading to close-to-zero posterior probability of belonging to Class 1 for most subjects.

See Appendix E of the Supplementary Material for diagnostics applied for D6N500 priors.

#### 6.2.3 Miniscule-class chains

**Figure 8:**
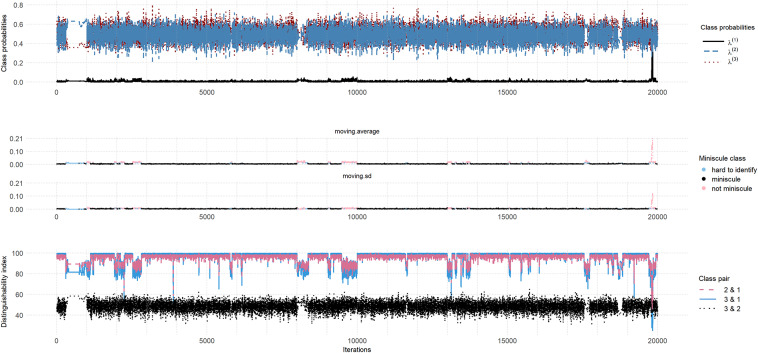
(Color online) Miniscule-class behavior for D2N500 priors. *Note*: Top Panel: Traceplot of 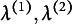 , and 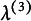 Mid Panel: Moving average and moving standard deviation of 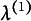 Bottom Panel: Distinguishability index for all class pairs.

The top panel of Figure 8 shows a traceplot of the latent class probabilities for the D2N500 priors. We found that three parameters have 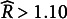 and the average 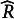 for all parameters is 1.051. Our stuck-sequence diagnostic identified 17 stuck sequences (6 in Chain 1, 2 in Chain 8, and 5 in Chain 18), all but one shorter than 40 and one in Chain 1 of length 353. The moving average and moving standard deviation plots in the middle panel of Figure 8 and the DI plot in the bottom panel of the figure suggest that miniscule-class behavior persists for entire chains and for long sequences in other chains.

## 7 Simulation study of strategies to detect and avoid problematic behavior

### 7.1 Simulation design

We simulated a new set of four responses for each of the 405 children in the NLSY data using parameter estimates from the well-behaved three-class solution with occasion number (0,1,2,3) as time variable. By simulating data instead of using real data, we can estimate the correct model so that we know that any degenerate nonidentifiability is not due to overfitting. Moreover, simulation ensures that our findings are not driven by some feature in the data (e.g., outliers) that we are unaware of.

The parameter values used for the simulation are in Sim_design.code.R and the code for simulating the data is in SimCode.source.R, both in the Simulation_study folder of the GitHub repository. Priors and Stan parameters were varied as described below, while keeping the dataset the same to reduce variability.

The priors for 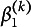 , 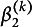 , 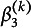 , 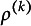 , and 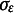 were as specified in Section 2.3.1, whereas the other priors were varied to discourage abnormal behavior to different degrees. One strategy to prevent miniscule-class and stuck-sequence behaviors is to discourage very small draws of 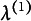 . This can be accomplished by using a weakly informative Dirichlet prior with concentration parameter 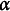 larger than two to steer the class probabilities toward equality (1/3 here). Three values of the concentration parameter were therefore considered, 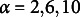 .

While specifying half-Cauchy distributions for standard deviation parameters is common practice in hierarchical models, this can lead to excessively large draws, such as 1,350,770 and 25,839,500 for the D2C5 priors, when 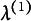 approaches zero. This, in turn, can cause the chain to become stuck, as observed in the persistently stuck example in Figure 6. Consequently, we recommend adopting half-normal priors for the random-intercept and random-slope standard deviations, with sufficiently large scale parameter 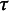 to avoid being overly informative but sufficiently small 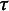 to avoid excessively large draws of the standard deviation parameters. For the simulation study, we compared the half-Cauchy distribution with scale parameter 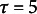 , denoted C5, with half-normal distributions with scale parameters 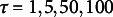 , denoted N1, N5, N50, and N100.

The Stan step size 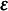 was set to 1 (the default) and to 0.01, using the argument step_size of the $sample method for CmdStan model objects.

For each combination of the three concentration parameters 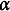 , five prior distributions/scale parameters 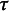 for the standard deviation parameters and two step sizes 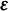 , we ran 100 chains on the same dataset. The chains can be viewed as replicates because they are independent.

We expect problematic behavior to be less likely as 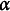 increases and as 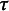 decreases, and generally less likely for a half-normal than a half-Cauchy distribution. For each value of 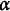 , we use the standard deviation priors in the order of “most difficult” to “least difficult”, i.e., C5, N100, N50, N5, and N1. If no problematic behavior is observed for N5, say, we do not consider the less difficult condition N1.

### 7.2 Simulation results

The step size specified for Stan did not appear to make any difference to the average step size used in the chains. The average step sizes used across the 100 chains for each condition ranged from 0.036 to 0.123, were sometimes larger when the small step size was specified than for the default for a given set of priors and never differed by more than 0.008 for a given set of priors. We also compared diagnostic results for small and default step sizes for each set of priors and found no systematic differences. We therefore pooled the results across step sizes, giving 200 chains per set of priors.

#### 7.2.1 Prevalence of problematic behaviors

Table 3 shows the impact of different priors on the prevalence of problematic behaviors according to our diagnostics and the prevalence of Stan warnings. The first two columns of Table 3 show the combinations of priors, while the other columns present the percentage of each problematic behavior or Stan warning. Except for 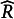 , the percentages are out of the 200 individual chains. Exact binomial 95% confidence intervals for these percentages have half-widths (approximate margins of error) of 3 percentage points when the point estimate is 5% or 95%, 6 percentage points when the point estimate is 20% or 80% and 7 percentage points when the point estimate is 50%. Since multiple chains are needed to compute the 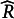 diagnostic and practitioners typically use 4 chains, the 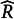 diagnostic was calculated as the proportion of fifty 4-chain batches with 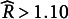 for at least one parameter.

**Table 3:** Percent problematic behavior according to different diagnostics and Stan warnings for different prior combinations

Priors		Proposed diagnostics					Stan warnings		
			Stuck			Stuck sequ.			Diverg.
		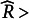	sequ.	Stuck	Miniscule	and/or		Max.	and/or max.
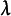	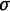	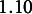	( 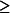 20 iter.)	chain	class	minisc. class	Diverg.	treedepth	treedepth
D2	C5	84.0	37.0	36.5	17.5	54.5	73.0	41.0	74.5
	N100	92.0	1.5	0.0	98.5	98.5	15.0	6.0	16.0
	N50	96.0	3.5	0.0	95.5	95.5	11.0	9.0	20.0
	N5	96.0	3.5	0.0	20.0	20.0	12.0	16.0	26.0
	N1	10.0	0.0	0.0	11.0	11.0	18.0	1.0	18.0
D6	C5	50.0	11.5	11.0	0.5	12.0	17.0	6.0	19.5
	N100	86.0	6.5	0.0	40.0	41.0	4.0	14.0	20.5
	N50	50.0	3.0	0.0	12.5	13.0	1.0	14.0	17.5
	N5	0.0	0.0	0.0	0.0	0.0	0.0	0.0	0.0
D10	C5	22.0	4.5	4.5	0.0	4.5	10.0	6.0	8.5
	N100	44.0	4.0	0.0	14.5	15.0	1.0	13.0	12.0
	N50	0.0	0.0	0.0	0.0	0.0	0.0	5.0	3.0

*Note*: D2, D6, and D10 are Dirichlet ( 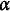 , 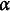 , 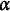 ) priors for ( 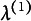 , 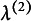 , 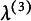 ), with 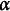 equal to 2, 6, and 10, respectively. C5 is a half-Cauchy(5) prior and N100, N50, N5, and N1 are half-normal priors for 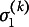 and 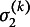 , with 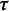 equal to 100, 50, 5, and 1, respectively. The other priors are given in Section 2.3.1.

A stuck sequence was defined as no change in 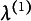 for at least 20 consecutive iterations, i.e., a moving standard deviation with window-size 10 being 0 for at least 11 consecutive iterations. A special case of a stuck sequence is a (persistently) stuck *chain* that is stuck for all iterations. A chain was classified as exhibiting miniscule-class behavior if the DI was greater than 95 in at least three consecutive iterations, excluding persistently stuck chains. The seventh column shows the percentage of stuck chains and/or minuscule-class occurrences.

Regarding twinlike-class behavior, we observed that across the 200,000 iterations for each set of priors, the DI was only occasionally smaller than five. This occurred twice (0.001% of iterations) for the D6N100 priors (smallest DI is 2.17), eight times (0.004% of iterations) for the D10C5 (smallest DI is 1.51), and seven times (0.0035% of iterations) for D10N100 (smallest DI is 1.97). However, these smallest DI values only occur for a single iteration, with neighboring iterations having larger values ranging from 14.01 to 65.41. Therefore, there is no evidence for self-perpetuating twinlike-class behavior. To create a situation where twinlike-class behavior is likely to occur, we simulated data from a two-class model and deliberately overfitted the data using a three-class model. See Appendix F of the Supplementary Material for more information and a graph showing two kinds of twinlike-class behavior (Class 1 of the data-generating model being represented by twins versus Class 2 being represented by twins), each persisting for an entire chain. This is a good example of degenerate nonidentifiability.

**Figure 9:**
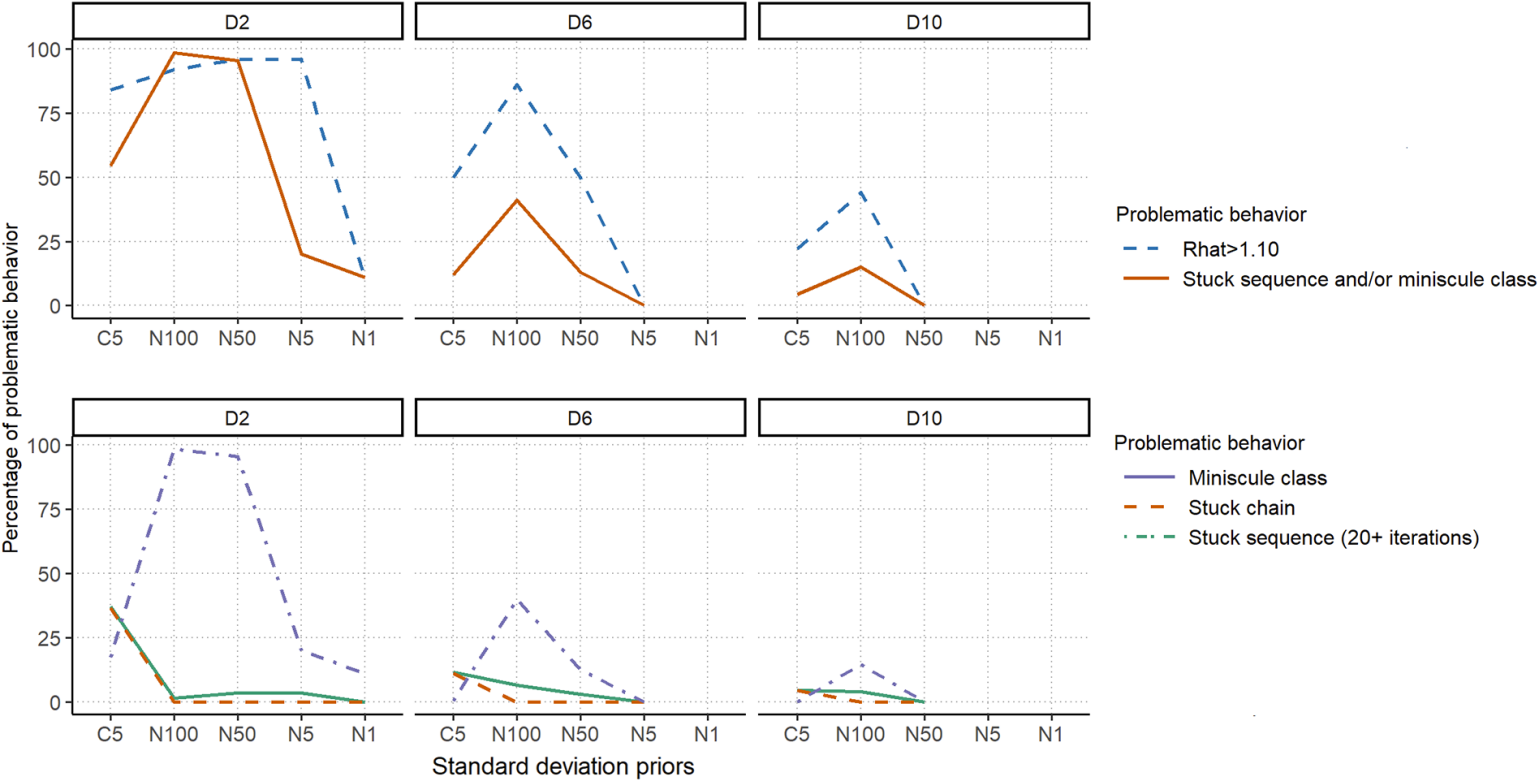
(Color online) Percent problematic behavior for combinations of standard deviation priors (*x*-axis) and Dirichlet priors (D2, D6, D10 from left to right). *Note*: Top panels: percentage of 4-chain batches with 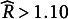 for any parameter (dashed) and percentage of chains with stuck-sequence and/or miniscule class behavior (solid). Bottom panels: percentage of chains with miniscule class (solid), stuck chain (dashed), and stuck sequence (dot-dash).

Figure 9 gives a visual representation of some of the simulation results, with standard deviation priors represented on the x-axis, Dirichlet priors in the columns, and different types of behaviors in the rows. In the top row, the dashed lines show the percentage of 4-chain batches with 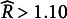 for at least one parameter. For half-normal distributions, this problem becomes rarer as 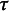 decreases and is more prevalent for smaller 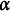 values. Specifically, 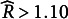 never occurred at 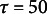 for 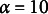 (D10N50) and at 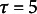 for 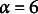 (D6N5), but occurred occasionally even at 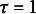 for 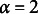 (D2N1). The solid lines in the top panel show the percent stuck-sequence (including stuck-chain) and/or minuscule-class behavior. Similar to large 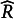 , this behavior becomes less frequent as 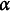 increases and 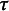 decreases.

As seen in the bottom row, persistently stuck chains (dashed lines) occur only for the half-Cauchy distribution, and this problem persists when the concentration parameter is as large as 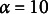 . Stuck-sequence (dot-dash lines) and miniscule-class (solid lines) behaviors become less likely as 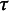 decreases for the half-normal distribution and as 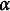 increases.

#### 7.2.2 Performance of proposed diagnostics

To assess the performance of the DI index in detecting minuscule-class behavior, we examined the 200,000 iterations for the D2N100 and D6N50 priors in more detail because this behavior was prevalent for D2N100 (98.5% of chains) and occurred infrequently for D6N50 (12.5% of chains).

For D2N100, the minimum, mean, and maximum values for 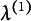 were <0.00, <0.00, and 0.04, respectively for the iterations where DI > 95. Conversely, for DI < 20, the values were 0.01, 0.16, and 0.29, with values below 0.04 occurring less than 2% of the time. We used receiver operating characteristic (ROC) curves to investigate the diagnostic accuracy of the DI for detecting 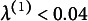 . The area under the curve (AUC) was 0.99. Using a threshold of DI > 95 gave a sensitivity of 0.52 and a specificity of 1.00. Using this threshold to detect 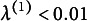 gave a sensitivity of 0.79 and specificity of 0.92. For D6N50, the minimum, mean, and maximum values for 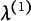 were <0.00, 0.01, and 0.05, respectively when DI > 95 and 0.22, 0.19, and 0.45 when DI < 20.

These results for D2N100 and D6N50 show that DI > 95 is associated with very small 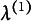 , irrespective of the frequency of minuscule-class behavior. The specificity is therefore high for detecting 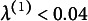 or 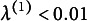 based on DI>95 in a given iteration. Regarding sensitivity, small values of 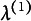 do sometimes occur when the DI < 20, and sensitivities for detecting 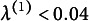 or 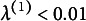 based on DI > 95 are modest. However, given that miniscule-class behavior often occurs several times in a chain and for long sequences of iterations, the sensitivity for detecting at least one occurrence of miniscule-class behavior in the chain should be high.

#### 7.2.3 Performance of Stan warnings

The final three columns of Table 3 present the percentage of chains in which Stan flagged at least once that there was a divergent iteration or that the maximum treedepth limit of 10 was reached. Reassuringly, these warnings occurred rarely for D6N5 and D10N50 priors that exhibited none of the problem behaviors considered. We now investigate the diagnostic accuracy of these Stan warnings for the problem behaviors considered in this article.

First, for diagnosing stuck chains, we consider the D2C5 priors, where the problem behavior occurred most frequently across the 200 chains (36.5% of the time). The ROC curves for diagnosing stuck chains, based on the number of divergent transitions per chain or the number of times the maximum treedepth was reached, showed high diagnostic accuracy, with AUCs of 0.99. For example, diagnosing a chain as stuck when the number of divergent transitions per chain exceeded 90 achieved a sensitivity of 0.98 and a specificity of 0.95. Similarly, basing the diagnosis on at least one occurrence of maximum treedepth reached resulted in perfect sensitivity (1.00) and a specificity of 0.94. As pointed out earlier, large 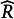 approaching infinity and tiny ESS near zero are also excellent diagnostics.

Second, for diagnosing stuck sequences of length 20 or longer (that are not stuck chains), we considered the D6N100 priors where this behavior occurred most often (6.5% of the time), excluding half-Cauchy conditions where the stuck sequences were stuck chains. The Stan warnings were not useful for detecting this problem, with AUCs of 0.51 and 0.41 for divergent transitions and maximum treedepth, respectively.

We also assessed Stan warnings for detecting minuscule-class behavior, choosing the D6N100 priors for which the prevalence of this behavior is 40%, suitable for obtaining good estimates of both sensitivity and specificity. The AUCs for the number of divergent transitions per chain and the number of times the maximum treedepth was reached were 0.53 and 0.37, respectively, suggesting that diagnostic accuracy is no better than chance.

Finally, the E-BFMI computed by Stan is rarely below the threshold of 0.3 across the twelve simulation conditions. This Stan warning occurs in only one chain (0.5% of the chains) for conditions D2N5 and D2N1 and five times (2.5% of the chains) for D6N100 (not shown in the table). This warning does therefore not appear to be useful for diagnosing the problem behaviors considered.

## 8 Concluding remarks

Inspired by challenges faced when applying Bayesian GMMs to reading recognition data from the NLSY, we have investigated identifiability issues that can lead to problematic estimation behavior.

In addition to the problems uncovered here, local maxima in both the likelihood function and posterior can cause MCMC chains to get trapped near a mode or move between modes, with proportions of iterations spent in each mode not representing their weights in the posterior (Yao et al., 2022). If the modes represent qualitatively different solutions, posterior means will no longer be meaningful. As discussed in Section 3.3 and demonstrated in Appendix F of the Supplementary Material, such modes can correspond to distinct but (nearly) observationally equivalent ways of degenerating to a solution with fewer classes.

We have also not discussed problems associated with estimating generalized linear mixed models (GLMMs) which clearly also affect GMMs because they are finite mixtures of GLMMs. For example, GLMMs suffer from estimates of variance–covariance parameters being on the boundary of the parameter space. While McNeish & Harring (2020) address this problem of GMMs by specifying marginal covariance structures directly instead of using random effects, another approach would be to use weakly informative priors that nudge estimates away from the boundary (e.g., Chung et al., 2013). Constraining covariance matrices to be equal across classes, 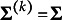 , also mitigates this problem. In addition, this constraint ensures that the data provide information on the variance–covariance matrix even if some class-probabilities are zero, thereby making such (near)-degenerate sampling less self-perpetuating. However, setting the matrices equal can rarely be justified by subject matter arguments.

Finally, we have not investigated the robustness concerns raised by Bauer & Curran (2003), Hipp & Bauer (2006) and Bauer (2007), among others. For instance, Bauer & Curran (2003) point out that a univariate finite mixture of normal densities is often used to approximate a non-normal distribution, highlighting that mixture components can merely serve the purpose of relaxing distributional assumptions and need not correspond to distinct subpopulations.

In this article, we have argued that likelihood identifiability is the basis for Bayesian identifiability and that the marginal likelihood should be considered in hierarchical models. Finite mixture models suffer from several kinds of identifiability problems. One of them is that the models are not locally identified at degenerate parameter points. Whereas other researchers have discussed the problem of such degeneracies for true model parameters or for parameter estimates, we are not aware of previous work pointing out the problems that occur when posterior draws of parameters are degenerate or near-degenerate. When near-degeneracy is due to one or more class probabilities being close to zero, we have “self-perpetuating near-degenerate draws” that manifest as stuck sequences, stuck chains, and what we call miniscule-class behavior, where the class probability parameter(s) fluctuate around a mean close to zero for sequences of iterations or for the entire chain.

A simulation study showed that problematic behavior is quite common when vague priors are used for the latent class probabilities (e.g., Dirichlet with concentration parameter 2) and for random-intercept and random-slope standard deviations (e.g., half-Cauchy with scale parameter 5). As pointed out by a reviewer, this behavior may be more detectable with HMC than with conventional sampling methods such as Metropolis and Gibbs sampling because HMC tends to be better at exploring the posterior in a given number of iterations. For our simulated dataset, larger concentration parameters (such as 6 or 10) and half-normal instead of half-Cauchy priors for standard deviations (with scale parameter 50 or smaller), largely prevented problematic behavior.

We recommend against specifying half-Cauchy priors for standard deviation parameters in GMMs because this often leads to stuck-sequence and miniscule-class behavior. Instead, somewhat informative half-normal priors can be used. Unfortunately, we cannot recommend specific values for the scale parameter because what works best will depend on the sample size and the units of measurement of the variables entering the model (e.g., time measured in days versus years).

The Dirichlet prior for the class probabilities should also be made somewhat informative, by choosing a sufficiently large concentration parameter 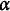 . A good strategy would be to start with a small value for 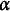 and perform diagnostics for problematic behavior. Then, if necessary, increase the concentration parameter. However, if miniscule class behavior is replaced by twinlike behavior when 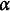 is increased, this may indicate that the number of classes specified is greater than the actual number of classes (see also Appendix F of the Supplementary Material). As shown by Rousseau & Mengersen (2011) for a general class of finite mixture models, such overfitting asymptotically results in classes being empty for 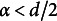 and classes merging for large 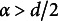 , where *d* is the number of class-specific parameters.

It is important not to make the priors excessively informative to avoid undue influence of priors on the final estimates, which can be assessed using sensitivity analyses (e.g., Depaoli et al., 2017).

Several diagnostics were proposed for detecting problematic behavior. While 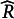 and ESS from the posterior package, as well as Stan warnings regarding maximum treedepth and divergent transitions can be indicative of completely stuck chains, these diagnostics cannot be used reliably to detect shorter stuck sequences. The moving standard deviation is designed for this purpose, and we suggest using it to detect stuck sequences. We also recommend computing our proposed DI and inspecting a traceplot of this index. Although we designed the DI for detecting twinlike behavior (when it is close to 0), it is also powerful for detecting miniscule-class behavior (when it is close to 100). A traceplot of the smallest class probability, along with its moving standard deviation are also useful graphs for seeing the miniscule-class sequences. We hope that the code provided on https://github.com/DoriaXiao/BayesianIdentification will facilitate use of these diagnostics.

## Supporting information

Xiao et al. supplementary material Xiao et al. supplementary material

## Supplementary material

The supplementary material for this article can be found at https://doi.org/10.1017/psy.2025.11.

## A Identifiability in hierarchical models

Swartz et al. (2004, p.3) consider the model

where  is known. Swartz et al. (2004, p.3) point out that the model is not likelihood identified even though “there is no practical problem with this model” in that the marginal posterior means of  and  depend on the data. The reason for this apparent contradiction is that they consider the likelihood  for which  does not imply that  .

However, we argue that the relevant likelihood is the *marginal* likelihood  , which is a normal density function with mean  and variance 2. The corresponding prior  is a normal density with known mean  and variance 1. Based on the marginal likelihood  , the model is identified.

Alternatively, if we want to condition on  in the likelihood, we can marginalize the prior over  , i.e., use the likelihood  , a normal density with mean  and variance 1, and define the prior as  , a normal density with mean  and variance 2.

## B Stan code for GMM

Equation (9) defined the fully marginal likelihood as

and the corresponding log-likelihood can be written as

(10)

The full Stan code is given below. The steps for evaluating the log-likelihood are:

First,  for each subject *j* is computed in the mmn function, defined in the functions block (lines 1–14). The arguments of this function are the parameters for a class *k* and the data for a subject *j*, extracted using the segment function (lines 75 and 76) within the transformed parameters block.

Second, we adapt the finite mixture model example from the Stan User’s Guide [73] to our specific model. Instead of declaring one local array variable lps of size *K*, as done in the User’s Guide, we declare a *J*-array of lps of size *K* (line 64) within the transformed parameters block. The *K*-dimensional vector lps[j] has *k*th element equal to  , the log contribution from the *k*th mixture component for the *j*th subject (lines 71–78).

Lastly, the log_sum_exp(lps[j]) function computes the logarithm of the sum of the exponentiated elements of lps[j], thereby aggregating the likelihood across all classes for each subject *j*. The marginal log likelihoods are then added directly to the log posterior via the target += statement (line 99).
