# Supplementary material for: Bayesian Identification and Estimation of Growth Mixture Models
Source: Psychometrika. 2025 Apr 7;90(2):442–75. doi: 10.1017/psy.2025.11 (PMC12483708; doi:10.1017/psy.2025.11)
Supplement: Xiao et al. supplementary material [file S0033312325000110sup001.pdf]

Supplementary Material for
Bayesian Identification and Estimation of Growth Mixture Models
Xingyao Xiao, Sophia Rabe-Hesketh, Anders Skrondal
DOI: 10.1017/psy.2025.11

Web Appendix C: Further results for Section 2.4

Results for occasion number as time variable

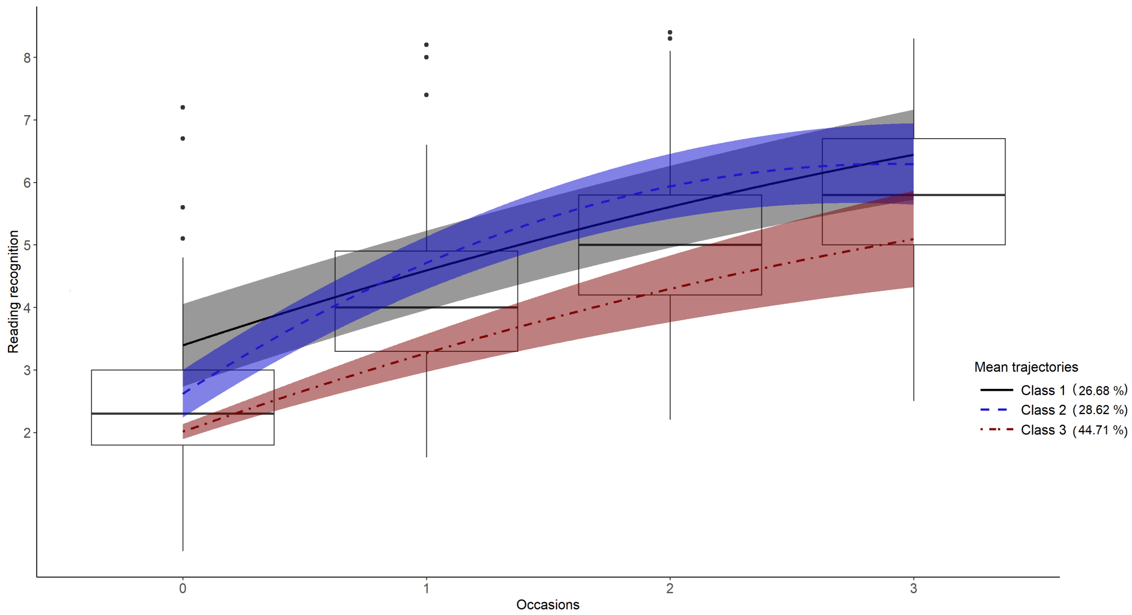

Figure 10: Class-specific mean trajectories with shaded 50% mid-range and box-plots of reading scores

Table 1: Information criteria for GMMs with 1 to 4 classes for D10N50 priors with occasion number as time variable (lowest value for each criterion in italics)

	Number of classes			
Information criterion	1	2	3	4
WAIC	3189.54	3105.20	*3088.72*	3102.28
−2ELPD_LOO	3189.58	3105.24	*3088.86*	3092.26

Table 2: Estimates for D10N50 priors with occasion number as time scale

Class	Parameter	Posterior mean	Posterior SD	Cred. interval 2.50%	97.50%	$\widehat{R}$
	$\lambda^{(1)}$	0.267	0.062	0.154	0.394	1.005
	$\beta_1^{(1)}$	3.393	0.220	3.024	3.893	1.002
	$\beta_2^{(1)}$	1.290	0.185	0.903	1.639	1.003
1	$\beta_3^{(1)}$	-0.091	0.052	-0.191	0.018	1.002
	$\sigma_1^{(1)}$	0.979	0.121	0.757	1.239	1.002
	$\sigma_2^{(1)}$	0.262	0.060	0.142	0.381	1.001
	$\rho^{(1)}$	-0.566	0.194	-0.860	-0.117	1.001
	$\lambda^{(2)}$	0.286	0.070	0.154	0.428	1.003
	$\beta_1^{(2)}$	2.618	0.135	2.352	2.896	1.002
	$\beta_2^{(2)}$	2.528	0.206	2.186	3.000	1.002
2	$\beta_3^{(2)}$	-0.434	0.065	-0.584	-0.327	1.003
	$\sigma_1^{(2)}$	0.564	0.108	0.337	0.755	1.002
	$\sigma_2^{(2)}$	0.257	0.057	0.145	0.370	1.002
	$\rho^{(2)}$	0.042	-0.276	0.440	0.631	1.001
	$\lambda^{(3)}$	0.447	0.054	0.343	0.555	1.003
	$\beta_1^{(3)}$	2.013	0.052	1.912	2.115	1.001
	$\beta_2^{(3)}$	1.373	0.108	1.148	1.569	1.004
3	$\beta_3^{(3)}$	-0.116	0.028	-0.168	-0.058	1.003
	$\sigma_1^{(3)}$	0.179	0.060	0.057	0.296	1.005
	$\sigma_2^{(3)}$	0.357	0.040	0.277	0.434	1.000
	$\rho^{(3)}$	0.666	0.204	0.205	0.954	1.002
	σ_e	0.468	0.016	0.438	0.501	1.000

Web Appendix D: Example of somewhat indistinguishable classes

In Chain 5 with D4N100 priors, the posterior means of the class-specific parameters for classes 1 and 2 are very similar. For comparison, we also present an example of normal behavior using Chain 5 with D10N50 priors; see Figures 11a and 11b.

We found that the ESS of the class probabilities were a little lower for the D4N100 priors when the class 1 and 2 were less distinguishable (282, 247, and 332 for $\lambda^{(1)}$ to $\lambda^{(3)}$) than for the D10N500 priors (313, 321, and 508, respectively).

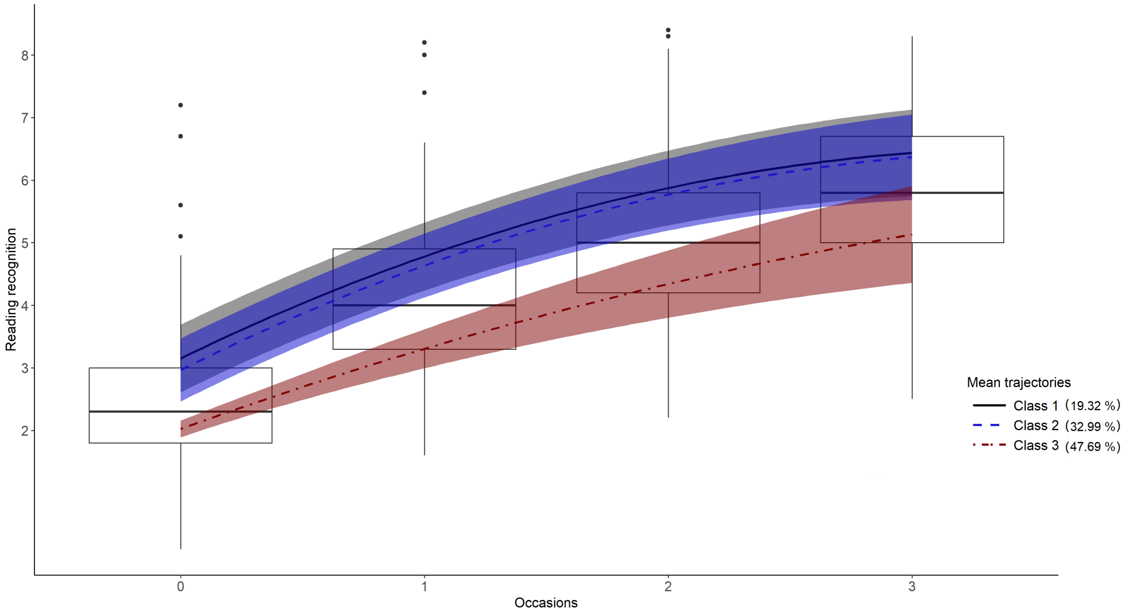

(a) Example of indistinguishable classes: Chain 5 with D4N100 priors

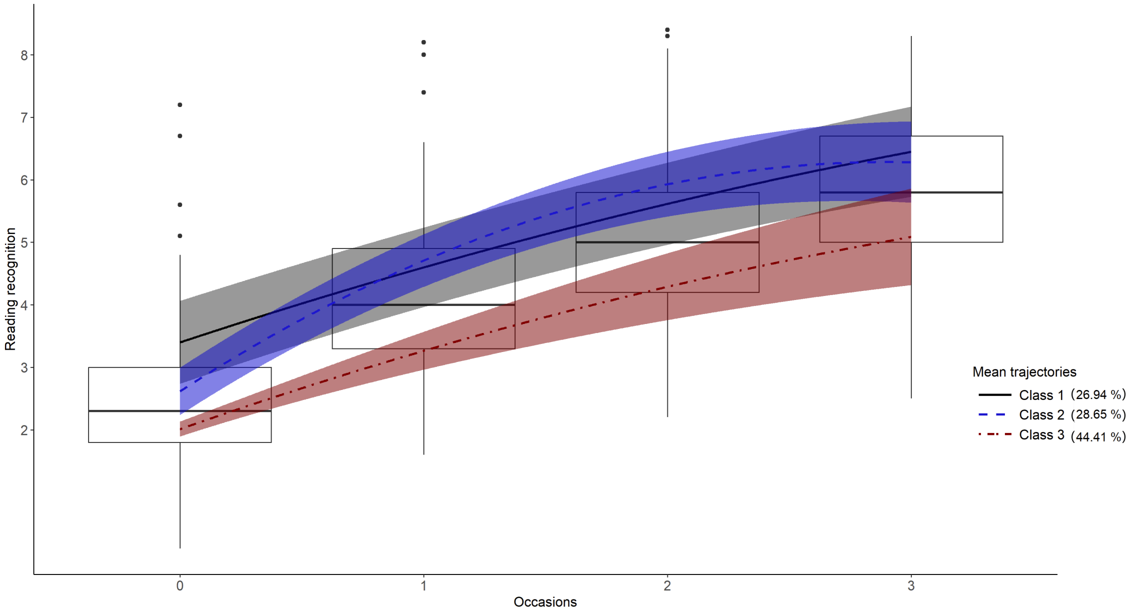

(b) Example of normal behavior: Chain 5 with D10N50 priors

Figure 11: Class-specific mean trajectories with shaded 50% mid-range and box-plots of reading scores

Web Appendix E: Example of miniscule-class behavior

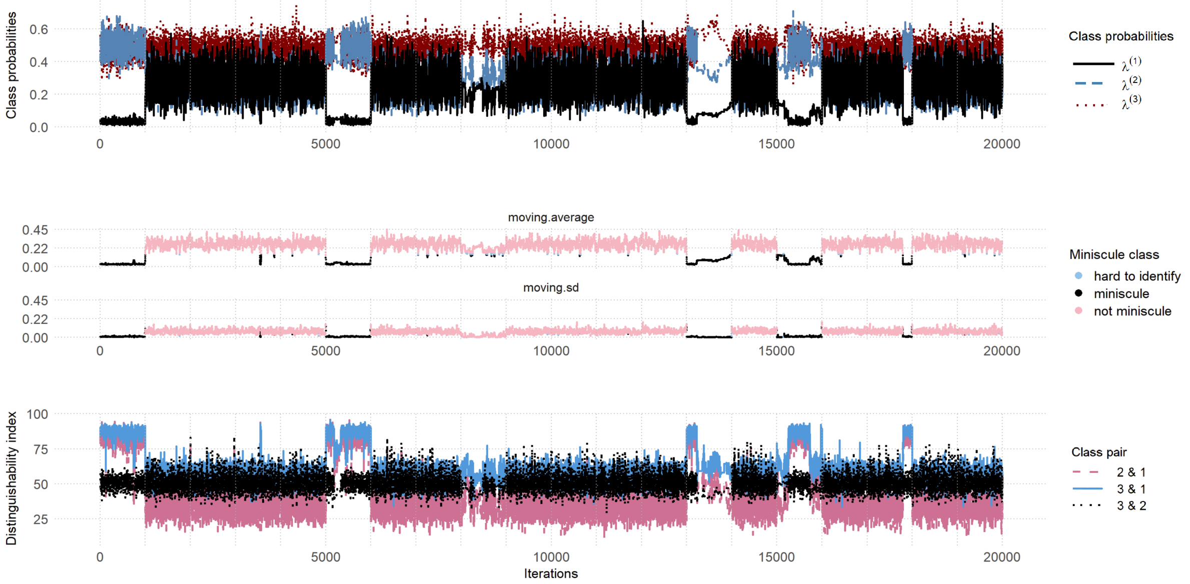

Top Panel: Traceplot of $\lambda^{(1)}$, $\lambda^{(2)}$, and $\lambda^{(3)}$
Mid Panel: Moving average and standard deviation of $\lambda^{(1)}$
Bottom Panel: Distinguishability index for all class pairs

Figure 12: Miniscule-class behavior for D6N500 priors

Web Appendix F: Twinlike-class behavior due to specifying too many classes

We simulated data for 400 subjects (level-2 sample size), with each subject observed at 5 time points, resulting in a total level-1 sample size of 2,000 observations. The timing of the 5 time points, $t_{ij} = i - 1$, ranges from 0 to 4. The data-generating model is a two-class GMM with well-separated classes. Specifically, the mean intercepts $\beta_1^{(1)} = 6$ and $\beta_1^{(2)} = 10$ are separated by $5\sigma_1^{(1)}$, where $\sigma_1^{(1)} = 0.8$ is the larger random-intercept standard deviation among the two classes, and the mean slopes $\beta_2^{(1)} = 0.3$ and $\beta_2^{(2)} = 2.7$ are separated by $4\sigma_2^{(1)}$, where $\sigma_2^{(1)} = 0.6$ is the larger random slope standard deviation. The class-2 random intercept and slope standard deviations are $\sigma_1^{(2)} = 0.5$ and $\sigma_2^{(2)} = 0.3$. Additionally, the correlations between the random intercepts and slopes are $\rho^{(1)} = 0.6$ and $\rho^{(2)} = 0.3$. The level-1 residual standard deviation is constant across classes at $\sigma_e = 1$, resulting in an intraclass correlation of 0.88 at the last time point. The class probabilities are $\lambda^{(1)} = 0.2$ and $\lambda^{(2)} = 0.8$.

Following our recommendation, we used the Dirichlet prior with a large concentration parameter of 10 for the class probabilities and a half-normal prior with a scale parameter of 10 for random-intercept and random-slope standard deviations. The priors for other parameters are consistent with the choices in Section 2.3.1.

To illustrate the twinlike-class behavior, we deliberately overfitted the data by estimating a three-class GMM to this two-class data-generating model. We also estimated a two-class model for comparison. After a 1,000-iteration warmup, four chains were run with each chain having 1,000 iterations, none of which resembled a normal three-class solution. We then use the first two chains to exemplify the different degeneracies that can occur between chains, while noting that different degeneracies can also occur within a single chain.

Figure 13 displays the traceplot of $\lambda^{(1)}$, $\lambda^{(2)}$, and $\lambda^{(3)}$ from the three-class solution in the top panel and the corresponding pairwise distinguishability indices (DI) in the bottom panel (where iterations 1-1000 correspond to Chain 1 and iterations 1001-2000 to Chain 2). The DI plot indicates that classes 1 and 2 are nearly indistinguishable in Chain 1, while classes 2 and 3 are nearly indistinguishable in Chain 2, the DI for the twinned classes remaining at a lower level throughout for Chain 1 than for Chain 2. Figure 14 suggests that the twinned classes 1 and 2 in Chain 1 correspond to class 1 in Chain 2 because $\lambda^{(1)} + \lambda^{(2)}$ in Chain 1 has a similar traceplot as $\lambda^{(1)}$ in Chain 2 (black traceplots). We also see that class 3 in Chain 1 corresponds to twinned classes 2 and 3 in Chain 2 (red traceplots). Table 3 shows the posterior means and standard deviations for the traceplots in Figure 14 (Chain 1: $\lambda^{(1)} + \lambda^{(2)}$ and $\lambda^{(3)}$; Chain 2: $\lambda^{(1)}$ and $\lambda^{(2)} + \lambda^{(3)}$), and shows that they closely resemble the posterior means and standard deviations of the λs in the two-class solution. The mean deviances ($-2L$, averaged over sampled parameters) in Chains 1 and 2 are also close to the mean deviance of the two-class solution.

These findings suggest that three-class model degenerates in two different ways that are (nearly) observationally equivalent to the two-class solution, and that these degeneracies are local modes of the posterior. In Chain 1, the smaller class of the two-class solution (and data-generating model) is approximately duplicated (or represented by twinned classes), whereas the larger class is duplicated in Chain 2. Such a scenario of local maxima corresponding to approximately observationally equivalent degenerate solutions (degenerate nonidentifiability) was briefly discussed in Section 3.3. Such behavior could easily be missed, illustrating the utility of the DI and of looking at the mean deviance.

Interestingly, this twinlike behavior mirrors the asymptotic results of Rousseau and Mengersen (2011). For a general class of finite mixture models, they show that overfitting will asymptotically result in components merging (corresponding to our twinlike behavior) when the number of class-specific parameters ($d = 5$ in our case) is less than 2α. In contrast, when $d > 2\alpha$, components become empty.

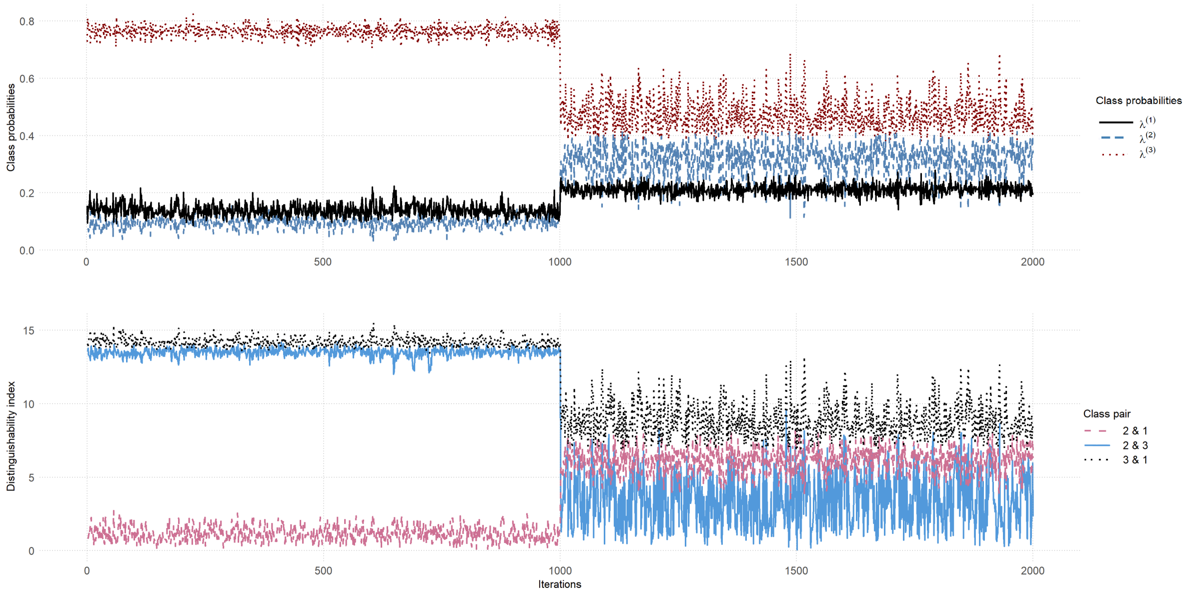

Top Panel: Traceplot of $\lambda^{(1)}$, $\lambda^{(2)}$, and $\lambda^{(3)}$
Bottom Panel: Distinguishability index for all class pairs

Figure 13: Twinlike-class behavior for the simulated two-class model across chains 1 and 2

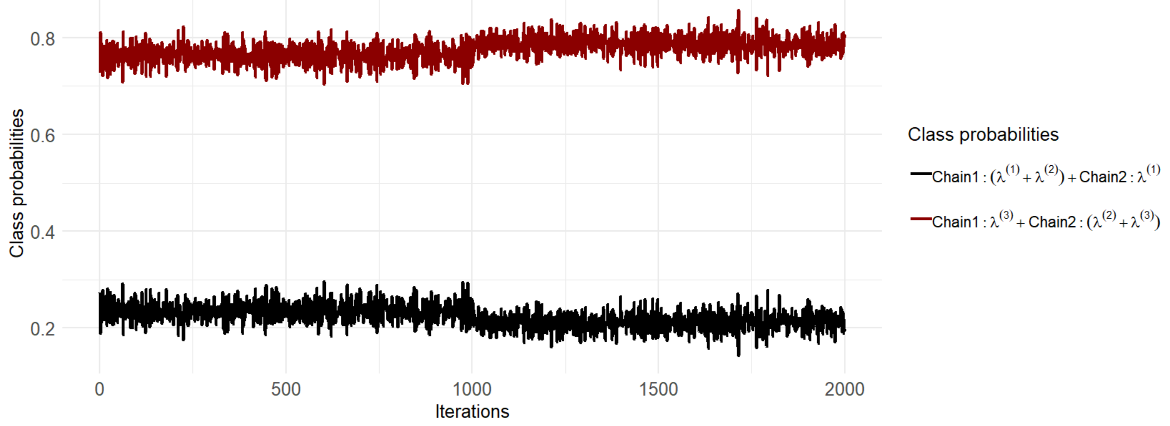

Figure 14: Traceplot of class probabilities when twinned classes (classes 1 and 2 in Chain 1 and Classes 2 and 3 in Chain 2) are combined

Table 3: Comparison of posterior summaries for two-class and three-class solutions

Number of classes	Chain	Posterior estimates of class probability			Deviance	
		λs or the addition of λs and the remaining λ	Mean	Standard deviation	Mean	Standard deviation
2	All four	$\lambda^{(1)}$	0.22	0.02	7141.77	5.16
		$\lambda^{(2)}$	0.78	0.02		
3	1	$\lambda^{(1)} + \lambda^{(2)}$	0.24	0.02	7145.57	6.56
		$\lambda^{(3)}$	0.76	0.02		
	2	$\lambda^{(1)}$	0.21	0.02	7144.47	6.02
		$\lambda^{(2)} + \lambda^{(3)}$	0.79	0.02		
